# Sustainable nanomedicine from olive leaf waste: biosynthesized ZnONPs with mechanistic validation against hepatocellular carcinoma

**DOI:** 10.1039/d6ra01605g

**Published:** 2026-10-06

**Authors:** Yasmin Mounir Mohamaden, Seham S. El-Hawary, Esmail M. El-Fakharany, Yousra A. El-Maradny, Mohamed El Raey, Amira Safwat El Senousy, Mohamed A. Rabeh, Mohammad D. AL-Zaid, Samar M. Bassam

**Affiliations:** a Department of Pharmacognosy, Faculty of Pharmacy, Cairo University Kasr El Aini 11562 Cairo Egypt yasmin.m.mohamaden@pharma.cu.edu.eg yasmin.m0unir.nm@gmail.com seham.elhawary@pharma.cu.edu.eg amira.elsenousy@pharma.cu.edu.eg; b Department of Pharmacognosy, College of Pharmacy, Arab Academy for Science, Technology and Maritime Transport (AASTMT) Alexandria Egypt yasmin.mounir@aast.edu; c Protein Research Department, Genetic Engineering and Biotechnology Research Institute (GEBRI), City of Scientific Research and Technological Applications (SRTA-City) New Borg El-Arab City 21934 Alexandria Egypt esmailelfakharany@yahoo.co.uk hiph.ymaradny@alexu.edu.eg; d Department of Phytochemistry and Plant Systematics, Pharmaceutical Division, National Research Centre Dokki 12622 Cairo Egypt elraiy@gmail.com; e Department of Pharmacognosy, College of Pharmacy, King Khalid University Abha 62251 Saudi Arabia mrabeh@kku.edu.sa; f Prince Faisal Bin Khalid Cardiac Centre - Aseer Cluster Abha 62521 Saudi Arabia Malziyd@gmail.com; g Department of Pharmacognosy and Natural Products, Faculty of Pharmacy, Pharos University in Alexandria Canal El Mahmoudia Street, Beside Green Plaza Complex 21648 Alexandria Egypt samar.bassam@pua.edu.eg

## Abstract

Agri-food waste is an underutilized source of valuable bioactive compounds. In this study, olive leaf pruning residues (*Olea europaea* L. *cv.* Soury) were used for the green synthesis of zinc oxide nanoparticles (OL-ZnONPs) as a natural reducing and stabilizing agent. Physicochemical characterization showed that the OL-ZnONPs had a hexagonal wurtzite structure, a crystallite size of approximately 15.5 nm, 82.8% crystallinity, and a zeta potential of −23.7 mV. TEM and SEM-EDX analyses revealed aggregated spherical particles with sizes of 4.15 ± 1.23 nm and 0.192 ± 0.067 µm, respectively, with zinc and oxygen accounting for 75.29% and 24.71%, respectively. LC-ESI-MS/MS-MRM analysis identified and quantified 11 phenolic compounds, with rutin (354.15 µg g^−1^), 3,4-dihydroxybenzoic acid (78.21 µg g^−1^), and quercetin (45.75 µg g^−1^) being the most abundant. OL-ZnONPs showed cytotoxic activity against HepG-2, MDA-MB-231, and HCT-116 cells, with the greatest selectivity toward HepG-2 cells (IC_50_ = 0.344 µg mL^−1^; SI = 5.279), compared with crude extract and 5-FU. OL-ZnONPs treatment increased intracellular levels of total caspase-3 and caspase-9, reduced TNF-α and IL-6 levels, induced nuclear changes consistent with apoptosis, and reduced HepG-2 cell migration. Network pharmacology was performed using the selected phenolic compounds together with ZnO to explore potential molecular mechanisms associated with the anti-hepatocellular carcinoma (HCC). A total of 801 anti-HCC-HepG-2 targets were predicted including AKT1, TP53, TNF, and IL6, CASP3 as hub genes in protein–protein interaction. The compound-target-pathway network identified quercetin, 3,4-dihydroxybenzoic acid, and ferulic acid as hub compounds. KEGG and GO enrichment analyses highlighted pathways in cancer, apoptosis, PI3K-Akt signaling, and chemical carcinogenesis-reactive oxygen species, providing exploratory mechanistic context for the experimental findings. Molecular docking predicted favorable binding of rutin and quercetin to AKT1. Collectively, these findings highlight the potential of OL-ZnONPs as an anticancer nanomaterial, particularly against HepG-2 cells, while supporting the valorization of olive leaf pruning residues aligns with the 2030 UN Sustainable Development Goal 3 (Good Health), SDG-9 (Industry/Innovation), SDG-12 (Responsible Consumption/Production), and SDG-13 (Climate Action). Further studies are required to clarify the contribution of the ZnO core and associated phytochemicals using size-matched bare ZnONPs, alongside *in vivo* evaluation of efficacy, biosafety, and pharmacokinetics.

## Abstract 2

Valorizing olive leaf waste into biosynthesized ZnONPs, this study demonstrates potent selective anticancer activity against HepG‑2 cells *via* apoptosis and migration inhibition, supported by network pharmacology aligning with UN SDGs.

## Graphical abstract

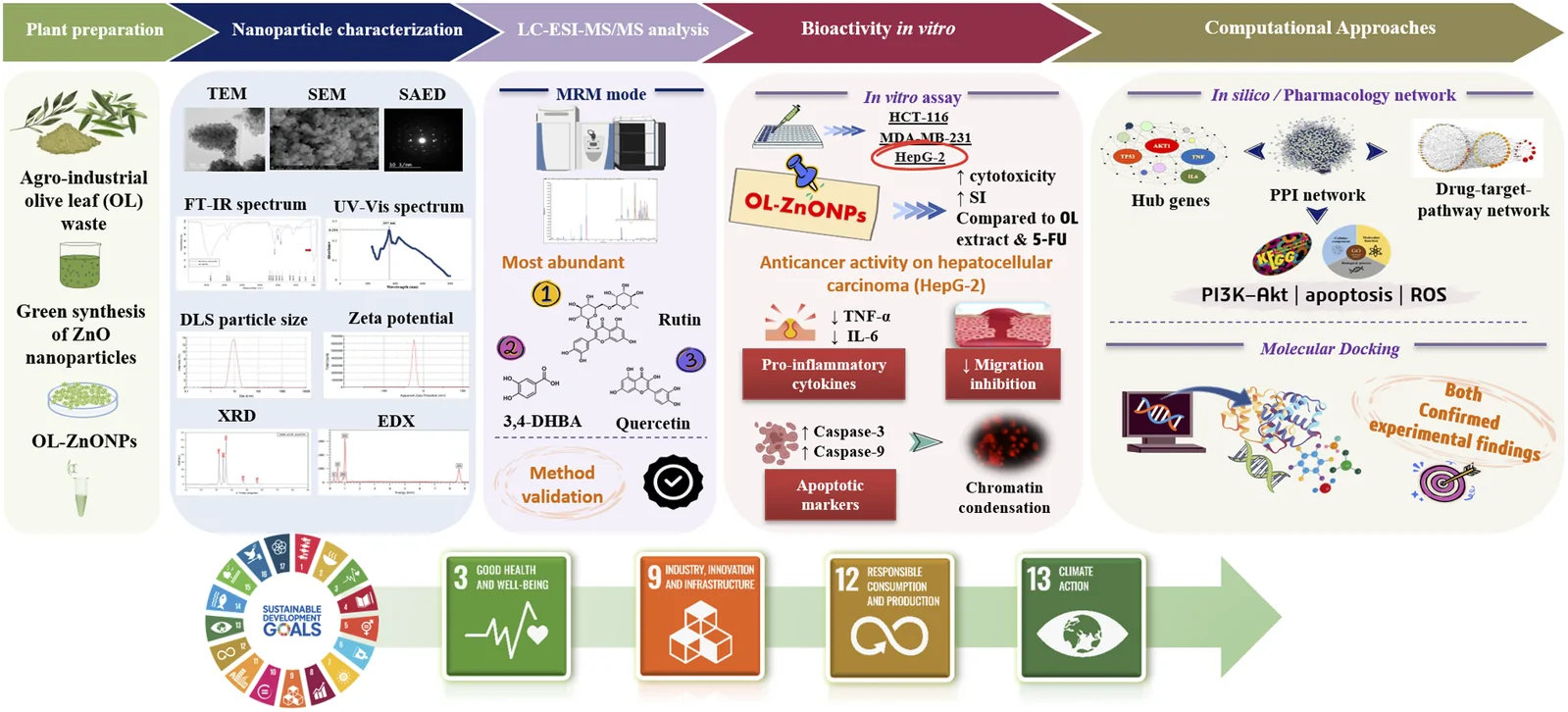

## 1 Introduction

ptCancer remains one of the leading causes of mortality worldwide, with liver, breast, and colon cancers representing major public health challenges. Liver cancer, in particular, is a significant global burden, ranking as the third leading cause of cancer-related deaths and the seventh most frequently diagnosed malignancy according to the GLOBOCAN 2024 database^1^ (Fig. S1a). Approximately 75–86% of primary liver cancers are hepatocellular carcinoma (HCC),^2^ whose incidence and mortality show marked geographic variation, with Asian countries reporting the highest rates due to regional etiological factors (Fig. S1b). Based on GLOBOCAN 2024 data released in 2026,^1,3^ 843 045 new cases of liver cancer were reported alongside 732 489 deaths, underscoring the close parallel between incidence and mortality (Fig. S1c and d). Projections suggest that annual cases may rise to 1.52 million by 2050 if current trends continue.^4^ Breast cancer is the second most commonly diagnosed malignancy worldwide, with 2.4 million new cases and 693 660 deaths reported in 2026,^1^ while colorectal cancer ranks third in incidence and second in mortality, accounting for nearly 10% of all cancer cases globally (Fig. S1).^1^

Despite advances in clinical management, therapeutic options for HCC remain limited. Only a minority of patients are eligible for curative interventions such as liver transplantation or surgical resection, which are restricted to early-stage diagnoses.^2^ Conventional treatments are further constrained by dose-limiting toxicities and drug resistance,^5^ highlighting the urgent need for innovative and sustainable strategies. Natural products are increasingly recognized as promising alternatives due to their structural diversity, biocompatibility, and potential multi-target pharmacological activities, in contrast to conventional single-target synthetic drugs.^6^

Among these natural resources, the olive tree (*Olea europaea* L., family Oleaceae) has long been used in traditional medicine across the Mediterranean and North Africa. Olive tree pruning generates nearly 25 kg of leaf biomass per tree annually, often considered agricultural waste.^7^ These leaves are rich in bioactive phytochemicals, including secoiridoids (oleuropein), simple phenols (tyrosol, hydroxytyrosol), flavonoids (quercetin, rutin), and triterpenes (oleanolic and maslinic acids), which collectively exhibit antioxidant, anti-inflammatory, hypocholesterolemic, antimicrobial, antihypertensive, hypoglycemic, and anticancer activities against diverse cancer cell lines.^8^

Zinc is an essential trace element and a cofactor for more than 300 mammalian enzymes involved in oxidative stress regulation, DNA synthesis and repair, cell cycle control, and apoptosis.^9^ Disruption of zinc homeostasis has been linked to cancer development, where reduced intracellular zinc promotes tumor initiation, while elevated concentrations exert cytotoxic effects.^9^ Building on these properties, zinc oxide nanoparticles (ZnONPs) have attracted considerable interest for biomedical applications, including antibacterial, antiviral, antifungal, wound-healing, antidiabetic, and anti-inflammatory applications.^10^ Their high surface area, chemical stability, and photocatalytic activity, combined with biocompatibility and biodegradability, have been associated with anticancer effects involving processes such as reactive oxygen species (ROS) generation and apoptosis induction.^10,11^

To enhance their therapeutic potential, recent research has emphasized green synthesis strategies that integrate natural products with nanotechnology. These eco-friendly approaches employ plant extracts as reducing and capping agents, offering sustainable alternatives to conventional chemical methods that are often costly and environmentally hazardous.^12^ Olive leaf (OL) extract, in particular, serves a dual role by reducing zinc ions and stabilizing the resulting nanoparticles. This approach offers a potential means of integrating the bioactive properties of olive phytochemicals with the physicochemical and biological properties of ZnONPs, providing a promising platform for sustainable nanomedicine.^13^

Recent studies have broadened the scope of green ZnO nanoparticle synthesis using diverse plant sources. Likewise, nanoparticle-based strategies for hepatocellular carcinoma therapy have gained momentum, with gold, silver, and ZnO nanoparticles shown to induce apoptosis and inhibit tumor growth in preclinical models.^14^ For instance, ZnO nanoparticles synthesized from *Echinops spinosus* root extract exhibited potent cytotoxicity against HepG-2 cells, showing 10.4-fold higher efficacy compared to crude extract, and moderate activity against MCF-7.^15^ Moreover, a chitosan/pomegranate peel extract/ZnO nanocomposite demonstrated cytotoxicity against HepG-2 cells with an IC_50_ of 129 µg mL^−1^, alongside notable antioxidant activity (DPPH scavenging 60.18%).^16^ Furthermore, ZnONPs synthesized from *Camellia sinensis* leaf extract reduced cell viability to 42% at 0.5 µg mL^−1^ and 27% at 5 µg mL^−1^ after 72 h.^17^ In addition, ZnO nanoparticles derived from *Eclipta prostrata* and *Pandanus odorifer* induced apoptosis in HepG-2 cells *via* caspase activation and DNA fragmentation.^11^

Although olive-mediated ZnONPs have been reported previously, most studies focused on basic characterization, antimicrobial and antioxidant activities, with limited mechanistic evaluation of anticancer potential, particularly in hepatocellular carcinoma (Table 1).^18^ Despite these advances, integrated frameworks combining eco-friendly synthesis, phytochemical profiling, mechanistic anticancer evaluation, and computational validation remain limited.

**Table 1:** Previous studies on olive leaf-derived nanoparticles and tested cancer cell lines

Part used for synthesis	Extraction solvent	Nanoparticle type	Cancer cell lines	Effects	Key reference
Leaves	Water	ZnONPs (green synthesis)	Breast cancer (MDA-MB-231)	Reduced cell viability at 24 h; 60% shrinkage, aggregation, and slowed proliferation	19
Leaves	Deionized water	Zn/Cu bimetallic nanocomposite	Lung cancer (SK-MES-1, and A-549, LK-2)	Showed significant anti-lung cancer activity with IC_50_ values of 154.00, 228.83, and 250.55 µg mL^−1^, respectively	20
Leaves	Deionized water	Oleuropein-loaded ZnO/Au mesoporous silica	Colorectal cancer (5-FU-resistant CRC cells)	Reduced cell viability and induced apoptosis in drug-resistant colorectal cancer cells	21
Leaves	Ethanol	ZnNPs, CuNPs, and AgNPs incorporated into a chitosan polymer	Breast cancer (MCF-7) and colorectal cancer (HCT-116)	All nanoparticle types reduced cell viability, induced morphological changes, and slowed proliferation, with ZnNPs showing minimal cytotoxicity toward normal cells	22
Leaves	Cold extraction using distilled water	AgNPs (green synthesis)	Human hepatocellular carcinoma (HepG-2), MCF-7, and human colorectal cancer (SW620)	Suppressed cell viability in these cancer cell lines	23
Leaves	Deionized water	ZnO nanorods, CeO_2_ nanorods (green synthesis, microwave-assisted)	Hepatocellular carcinoma cell line	The IC_50_ values were 117.24 µg mL^−1^ for ZnO and 103.50 µg mL^−1^ for CeO_2_	24

Building on this gap, this study aimed to combine sustainable nanotechnology with anticancer nanotechnology by valorizing olive leaf agricultural residues. Our systematic workflow included: [I] physicochemical characterization of biosynthesized OL-ZnONPs to confirm stability and structural properties; [II] exploration of associated phenolic acids and flavonoids using a validated LC-ESI-MS/MS-MRM method; [III] *in vitro* cytotoxicity assays on HepG-2, MDA, and HCT-116 cancer cell lines compared to normal cells and standard chemotherapy; and [IV] integration of experimental findings with computational approaches, including network pharmacology, KEGG pathway and GO enrichment analyses, and molecular docking to explore potential multi-component, multi-target molecular interactions and pathways.

By linking analytical precision with biological evaluation, this research highlights the potential of agri-food waste valorization for sustainable nanomaterial development. This integrated framework provides a basis for exploring potential molecular mechanisms and biological activities of OL-ZnONPs, while supporting the broader application of sustainable nanotechnology in alignment with the 2030 UN Sustainable Development Goals.

## 2 Materials and methods

### 2.1 Plant collection and extraction

Fresh olive leaves (*Olea europaea* L. *cv*. Soury) were collected in October 2022 from the Agricultural Research Station, Faculty of Agriculture, Cairo University, Egypt. The plant material was taxonomically authenticated by Dr Abdou Mohamed Abdallatif, Department of Pomology, Faculty of Agriculture, Cairo University. After thorough washing with tap water, the leaves were shade-dried, finely powdered, and stored in airtight, light-protected glass containers until use.

For extraction, 50 g of dried powdered leaves were macerated three times in 70% ethanol at room temperature. The combined filtrates were passed through Whatman No. 1 filter paper and concentrated under reduced pressure using a rotary evaporator (Heidolph, Germany) at 40 °C, yielding a dark green residue approximately 21.6 g of crude olive leaf extract.

### 2.2 Green synthesis of zinc oxide nanoparticles (ZnONPs)

Green synthesis of zinc oxide nanoparticles (ZnONPs) was synthesized using olive leaf (OL) extract according to the method described by,^25^ with minor modifications. Briefly, 1 g of dried OL extract was dissolved in 100 mL of ethanol and mixed with an aqueous solution of zinc acetate (5 g dissolved in 1000 mL of doubly distilled water). The mixture was heated in a water bath at 100 °C for 60 min. To initiate nanoparticle precipitation, 0.5 mL of 1 M ammonium hydroxide (Merck, Germany) was added dropwise while monitoring the pH at 12. The solution was left undisturbed for 20 min to ensure complete reduction of zinc acetate into ZnONPs. The resulting precipitate was centrifuged at 4000 rpm for 15 min, washed three times with ethanol and distilled water, and then freeze-dried to yield a pale green powder.

### 2.3 Characterization techniques of ZnONPs

#### 2.3.1 UV-visible spectrophotometry

The optical properties of the biosynthesized ZnONPs were examined using a UV-Vis spectrophotometer (Shimadzu UV-1800, Kyoto, Japan) to assess nanoparticle formation. Absorption spectra were recorded over a wavelength range of 200–800 nm at ambient temperature using a quartz cuvette with a 1 cm optical path length. The appearance of a characteristic absorption band support ZnONP formation.

#### 2.3.2 X-ray diffraction (XRD)

XRD provides information on crystalline structure, phase composition, lattice parameters, and crystallite size of ZnONPs on dried nanoparticles.^26^ Diffraction patterns were recorded with a Bruker D2 Phaser diffractometer (Bruker, Germany) equipped with a Cu Kα radiation source (*λ* = 1.5406 Å) over a 2*θ* range of 10°–90°. The average crystallite size was estimated using the Debye–Scherrer's equation from peak broadening, while Bragg's law was applied to calculate lattice spacing and assess the crystalline nature of the nanoparticles. As the technique is performed on dried powder samples, it yields statistically representative, volume-averaged values.

#### 2.3.3 Dynamic light scattering and zeta potential

The hydrodynamic diameter, particle size distribution, and surface charge of OL-ZnONPs were evaluated using a Zetasizer (Malvern Instruments Ltd). Dynamic light scattering (DLS) was applied to measure the hydrodynamic diameter (the core plus surrounding solvent layer), providing information on colloidal stability and size distribution through the polydispersity index (PDI). While electrophoretic light scattering (ELS) was employed to measure zeta potential and to evaluate colloidal stability.^27^

#### 2.3.4 Fourier transform infrared (FT-IR) spectroscopy

FT-IR spectroscopy was utilized to characterize phytochemical functional groups in the OL extract that contribute to the reduction and stabilization of ZnONPs. Characteristic moieties such as hydroxyl, carboxyl, and phenolic groups were identified, confirming their involvement in the green synthesis process.

Spectra of the dried OL extract and ZnONPs were recorded using a Bruker FT-IR spectrometer (UK) equipped with Tensor 37 software. Samples were prepared using potassium bromide (KBr) pellet method, and spectra were collected in the range of 4000–400 cm^−1^ under ambient conditions. This analysis revealed surface-associated organic residues, supporting the role of bioactive phytochemicals in nanoparticle formation.

#### 2.3.5 Transmission electron microscope (TEM) and selected area electron diffraction (SA.ED)

Transmission electron microscopy (TEM) was utilized to examine the actual particle size, morphology, and internal structure of biosynthesized ZnONPs. Micrographs were obtained using a JEM-2100 microscope (JEOL, Japan) operated at 200 kV accelerating voltage on dried nanoparticles. Particle size distribution was analyzed with ImageJ software, calibrated using scale bars for accuracy.

Selected area electron diffraction (SA.ED) patterns were also recorded to confirm the crystalline structure and lattice arrangement of the nanoparticles.

#### 2.3.6 Scanning electron microscope (S.EM) and energy-dispersive X-ray spectroscopy (EDX)

Scanning electron microscopy (S.EM) was employed to examine the morphology, particle distribution, and surface topology of OL-ZnONPs. Micrographs were obtained using a QUANTA FEG250 field emission SEM (FEI, USA) equipped with a backscattered-electron detector at 20 kV.

The elemental composition and purity of the nanoparticles were analyzed by energy dispersive X-ray spectroscopy (EDX) using a JEOL IT 200 system (JEOL Ltd, Japan). Particle size measurements were performed with ImageJ software, calibrated against scale bars to ensure precision and reproducibility.

### 2.4 Targeted LC-ESI-MS/MS approach in MRM mode

#### 2.4.1 Quantitative and qualitative profiling of phenolic and flavonoid compounds

A targeted LC-ESI-MS/MS analysis was performed to identify and quantify phytochemicals in the 70% ethanolic OL extract. The system operated in negative ionization multiple reaction monitoring (MRM) mode. Chromatographic separation was achieved on a Poroshell 120 EC-C18 column (3.0 × 100 mm, 2.7 µm) using an ExionLC™ AC system (SCIEX, USA). The mobile phase consisted of eluent A (0.1% formic acid in water) and eluent B (LC-grade acetonitrile), with a gradient elution program: 8% B (0–1 min), 15% B (1–4 min), 20% B (4–12 min), 30% B (12–20 min), and 45% B (20–25 min), followed by re-equilibration to 8% B (25.01–28 min). The flow rate was maintained at 0.4–0.5 mL min^−1^, with an injection volume of 5 µL.

Mass spectrometric analysis was conducted using a SCIEX Triple Quad 5500+ system (SCIEX, Singapore) equipped with an electrospray ionization source in negative mode. The instrument operated in MRM mode to ensure high sensitivity.^27^ Although secoiridoids such as oleuropein are well-documented as major constituents of olive leaves together with other related compounds,^28^ our LC-MS/MS-MRM analysis focused on phenolic acids and flavonoids due to the availability of 21 authentic standards. This selection was based on two main considerations. First, these compounds are widely reported to play direct roles in metal ion reduction (Zn^2+^) and nanoparticle stabilization, making them directly relevant to green synthesis of ZnONPs.^29^ Second, previous studies employing similar LC-MS/MS-MRM approaches with hot water and methanolic olive leaf extracts consistently identified rutin, quercetin, catechin, gallic acid, and related phenolics as dominant constituents, supporting the relevance of our targeted panel.^30,31^ This consistency supports the rationale for focusing on phenolic acids and flavonoids in the present study.

A total of 21 authentic standards were used for method validation and compound identification. Each analyte was identified by precursor/product ion transitions (Q1/Q3), retention time matching, and optimized MS parameters (decluttering potential, collision energy, and collision cell exit potential). Optimized MS conditions included: curtain gas 25 psi, collision gas 9 psi, ion source gases 1 and 2 at 50 psi, source temperature 500 °C, and ion spray voltage −4500 V. Detailed MRM transitions are provided in Table 2.

**Table 2:** Optimized MRM transitions and MS parameters for 21 phenolic acids and flavonoids in the 70% ethanolic olive leaf extract analyzed by LC-ESI-MS/MS^a^

No.	Compound name	MRM		Compound parameters		
		Q1 (*m*/*z*)	Q3 (*m*/*z*)	CE (V)	CXP(V)	DP(V)
1	Gallic acid	168.9	124.9	−30	−11	−110
		168.9	79	−30	−11	−110
2	Caffeic acid	178.9	135	−22	−9	−115
		178.9	107	−30	−7	−115
3	Rutin	609	299.9	−48	−15	−230
		609	270.9	−70	−9	−230
4	Coumaric acid	162.9	119	−20	−7	−90
		162.9	93	−40	−5	−90
5	Vanillin	151	136	−25	−9	−140
		151	92	−30	−7	−140
6	Naringenin	271.1	151	−30	−15	−130
		271.1	119	−40	−11	−130
7	Quercetin	301	151	−28	−9	−50
		301	178.8	−20	−7	−50
8	Ellagic acid	301	145	−40	−14	−120
		301	245	−38	−14	−120
9	3,4-Dihydroxybenzoic acid	152.9	109	−40	−5	−75
		152.9	90.9	−20	−7	−75
10	Hesperetin	301	164	−23	−10	−125
		301	136	−38	−10	−125
11	Cinnamic acid	146.9	102.6	−17	−6	−60
		146.9	77	−33	−6	−60
12	Methyl gallate	183	124	−30	−10	−110
		183	140	−30	−10	−110
13	Kaempferol	284.7	93	−46	−10	−120
		284.7	116.8	−52	−10	−120
14	Ferulic acid	192.8	133.9	−16	−5	−25
		192.8	177.9	−12	−5	−25
15	Syringic acid	196.9	122.8	−24	−5	−30
		196.9	181.9	−12	−5	−30
16	Apigenin	269	151	−15	−7	−35
		269	117	−15	−7	−35
17	Catechin	288.8	244.9	−16	−8	−40
		288.8	109	−32	−8	−40
18	Daidzein	253	132	−55	−10	−65
		253	91	−50	−13	−65
19	Chlorogenic acid	353	191	−35	−10	−60
		353	127	−40	−10	−60
20	Resveratrol	227	185	−26	−10	−53
		227	143.06	−40	−10	−53
21	Rosmarinic acid	359.1	161.03	−20	−10	−60
		359.1	197.05	−20	−10	−60

a CE, collision energy; CXP, collision cell exit potential; DP, decluttering potential.

#### 2.4.2 Calibration and linearity assessment

Calibration curves were constructed using five concentrations for each standard (0.01–1.0 µg g^−1^). Regression analysis was performed by plotting the peak areas against concentrations to determine slope, intercept, and coefficient of determination (*R*^2^), thereby assessing linearity.

#### 2.4.3 Sensitivity parameters: LOD and LOQ estimation

Limits of detection (LOD) and quantification (LO.Q) were calculated using the residual standard deviation-to-slope ratio method based on calibration data. In the absence of valid blank responses, the residual standard deviation (*σ*) from regression residuals was used to represent noise, while the slope (*S*) of the calibration curve was applied to calculate sensitivity parameters:LOD = 3.3 × (*σ*/*S*)LOQ = 10 × (*σ*/*S*)

This method, supported by,^32^ provided reliable estimates of analytical sensitivity and confirmed suitability for detecting low-abundance phenolic and flavonoid compounds.

### 2.5 Anti-cancer activity *in vitro*

#### 2.5.1 Cytotoxicity assessment

The cytotoxicity of OL-ZnONPs, OL extract, and 5-FU was assessed using the MTT assay.^33^ Human skin fibroblast (HSF) cells (1 × 10^3^ cells per well) were seeded in triplicate into 96-well plates containing Dulbecco's Modified Eagle Medium (DMEM; Elabscience, China) supplemented with 10% fetal bovine serum (FBS; Sigma-Aldrich, USA) and 1% penicillin/streptomycin. Cells were cultured until approximately 60% confluence. Cell lines were supplied by VACCERA, Egypt, and originally derived from the American Type Culture Collection (ATCC). Treatments with varying concentrations of OL-ZnONPs, OL extract, and 5-FU were applied, followed by incubation for 48 h at 37 °C under 5% CO_2_.

After treatment, the medium was removed, and cells were gently washed three times with phosphate-buffered saline (PBS). Then, MTT solution (200 µL, 0.5 mg mL^−1^) was added to each well, and plates were incubated for 3 h to allow formazan crystal formation. The solution was discarded, crystals were dissolved in 200 µL dimethyl sulfoxide (DMSO), and absorbance was recorded at 570 nm using a microplate reader. The half-maximal inhibitory concentration (IC_50_) values for 5-FU and nanoparticle formulations were determined using GraphPad Prism (version 9.0).

The anticancer activity was further evaluated against HepG-2 (human hepatoma), MDA (human breast cancer), and HCT-116 (human colon cancer) cell lines. Cells (1 × 10^3^ cells per well) were seeded in sterile 96-well plates in triplicate and allowed to reach approximately 60% confluence before treatment. IC_50_ values were subsequently determined relative to untreated controls, and selectivity indices (SI) were calculated to evaluate preferential cytotoxicity toward malignant cells. Morphological changes induced in cancer cells following treatments were observed using phase-contrast microscopy.^34^

#### 2.5.2 Quantification of pro-inflammatory and apoptosis-related markers

Pro-inflammatory and apoptosis-related markers were quantified in HepG-2 cells treated at their respective IC_50_ concentrations. Cells were lysed using radioimmunoprecipitation assay, and the total protein content was measured by the Bradford method with bovine serum albumin as the standard. The intracellular levels of total caspase-3, caspase-9, tumor necrosis factor-α (TNF-α), and interleukin-6 (IL-6) were measured using ELISA kits according to the manufacturer's instructions.^34^ Absorbance was recorded at 450 nm using a microplate reader.

#### 2.5.3 Apoptotic evaluation by nuclear staining

Apoptotic induction in HepG-2 cells was further assessed using nuclear staining. Cells were treated with IC_50_ concentrations of OL-ZnONPs, OL extract, and 5-FU for 48 h, followed by staining with propidium iodide (PI). Fluorescent images were captured using a phase-contrast fluorescence microscope (Olympus, Japan) to assess nuclear integrity and morphological features. Associated with apoptosis, including nuclear fragmentation and chromatin condensation.^35^

#### 2.5.4 Migration inhibition assay (scratch assay)

The anti-migratory activity of OL-ZnONPs, OL extract, and 5-FU was evaluated in HepG-2 cells using the scratch assay. Cells were cultured in 24-well plates until a uniform monolayer of approximately 90% confluence was established. To mimic a wound, a sterile pipette tip was used to create a linear scratch across the monolayer. The compounds were added at sub-IC_50_ concentrations (IC_50_/10) based on the cytotoxicity results. Scratch closure was monitored at 0, 24, and 48 h, and images of the scratch region were captured using a phase-contrast microscope to assess scratch closure and changes in cell migration following treatment.^35^

### 2.6 Network pharmacology

#### 2.6.1 Collection of active compounds

Compounds identified through MRM analysis were selected as the basis for network construction. In addition, ZnO was included as a separate component to assess its potential contribution to the biological activity of the green-synthesized ZnONPs and its possible relevance to hepatocellular carcinoma (HCC).

#### 2.6.2 Prediction of the compound-associated targets

Potential targets of the selected identified compounds were predicted using SwissTargetPrediction, BindingDB, SEA, HITP, and STITCH (accessed between November 18–20, 2025). Canonical SMILES of each compound were submitted with the organism set to *Homo-sapiens*. For BindingDB, the similarity threshold was set to 1 with no affinity restrictions. All retrieved protein targets were standardized and converted to official gene symbols using STRING (accessed on November 22, 2025) to ensure consistent annotation.^28^

#### 2.6.3 Screening and refinement of disease-related gene targets

Genes associated With hepatocellular carcinoma (HCC) were retrieved from DisGeNet, GeneCards, GEPIA, and OMIM (accessed September–November 2025) using multiple disease-related keywords, including “liver cancer”, “hepatocellular carcinoma”, “liver carcinoma”, and related synonyms. To ensure relevance to the experimental *in vitro* model, gene data specific to the HepG-2 cell line were also collected.

Intersection analysis was performed using Venny 2.0 (accessed November 23, 2025) to identify shared genes between HCC-associated targets and HepG-2-associated targets, referred to as the refined HCC-HepG-2 gene set (anti-HCC-HepG-2 targets). A second Venn comparison was then carried out between compound-associated targets and the refined gene set, and the overlapping genes were selected for subsequent PPI construction, network-based analyses, and enrichment analyses.

#### 2.6.4 Construction of the protein–protein interactions (PPI) network

The overlapping compound–disease targets were submitted to the STRING database (accessed November 23, 2025) with the organism set to *Homo sapiens* and a medium confidence score of 0.400. The resulting PPI network was imported into Cytoscape (version 3.7.2) to identify and visualize the top 20 hub genes ranked by degree centrality.^36^ Finally, the network was examined in the form of a directed graph with node size and color associated with a number of edges.

#### 2.6.5 KEGG pathway and gene ontology (GO) enrichment analysis

To elucidate the functional implications of the identified targets derived from MRM-identified compounds and ZnO against HCC-associated targets, Kyoto Encyclopedia of Genes and Genomes (KEGG) pathway enrichment analysis was conducted. Gene Ontology (GO) enrichment analysis annotations, including Cellular Component (CC), Molecular Function (MF), and Biological Process,^37^ were retrieved to categorize target functions and to generate visual representations of the top terms in each category, providing an overview of the multifunctional roles of bioactive-regulated targets. Analyses were restricted to *Homo sapiens*, using the default background.

The overlapping targets were uploaded into ShinyGO 0.80 to obtain enriched KEGG pathways and GO terms. Functional enrichment results were ranked according to −log_10_(FDR), and a false discovery rate (FDR) threshold of 0.05 was applied. The top 20 enriched terms were visualized using dot-plot charts.^38^

#### 2.6.6 Construction of the compound–disease–pathway network

Cytoscape software (version 3.7.2) was employed to construct and visualize the network comprising three components: selected compounds, their corresponding target genes (anti-HCC-HepG-2), and disease-related pathways. In this visualization, nodes represent compounds, targets, and pathways, while edges denote the interactions between these nodes.^28^ The network was refined by selecting the top nodes based on degree centrality, with node size and color used to visually emphasize the most influential components within the system.^38^

### 2.7 Molecular docking

Molecular docking was performed to investigate the interactions of top hub compounds identified from the compound–target–pathway network and LC-MS/MS profiling. Six predominant compounds were selected for analysis: rutin, 3,4-dihydroxybenzoic acid (3,4-DHBA), quercetin, chlorogenic acid, ferulic acid, and apigenin. These ligands were docked against key hub proteins, including serine/threonine kinase 1 (AKT1), TP53, and tumor necrosis factor-α (TNF-α), as well as apoptosis- and inflammation-related proteins examined *in vitro*: caspase-3 (CASP3), caspase-9 (CASP9), and interleukin-6 (IL-6).

#### 2.7.1 Receptor preparation

Protein structures in PDB format were downloaded from the Protein Data Bank. Crystallographic water molecules were removed prior to docking. Structural errors and missing atoms were corrected using PyMol, and binding regions were defined based on reported active site residues. Two-dimensional ligand structures were obtained from the PubChem database in SDF 2D format and converted to PDBQT format using Open Babel (version 2.4.1).

#### 2.7.2 Docking procedures

Docking poses and interaction maps were analyzed using ProteinPlus, and two-dimensional representations of protein–ligand interactions were generated with PoseView. This tool produces schematic diagrams of complexes with known three-dimensional structures, following standard chemical conventions. Intermolecular contacts were displayed using dashed lines to indicate directed interactions between ligand atoms and protein residues, while both components were rendered as structure diagrams. Hydrophobic interactions were represented using spline elements, represented as smooth curved lines that highlight hydrophobic ligand regions in proximity to corresponding protein residues. Diagram generation and layout handling are based on the 2Ddraw library.^39^ Binding affinity energies were calculated using AutoDock Vina, and docking scores were reported as described in.^40^

### 2.8 Statistical analysis

All experiments were performed in triplicate, and data are expressed as mean ± standard deviation. Statistical analysis was conducted using one-way ANOVA followed by Tukey's multiple comparison test in GraphPad Prism (version 9). Differences were considered statistically significant at *p* < 0.05.

## 3 Results and discussion

### 3.1 Physical characterization of ZnONPs

#### 3.1.1 UV-visible spectrophotometry

UV-visible spectroscopy was employed as a preliminary optical analysis to confirm the formation of the green-synthesized ZnONPs. The absorption spectrum recorded in the range of 200–800 nm revealed a distinct peak at 357 nm with an absorbance value of 0.254, which was indicative of nanoparticle formation (Fig. 1a). This observation is consistent with earlier research that reported a characteristic absorption peak at 370 nm for ZnONPs synthesized using an ethanolic extract of *Olea europaea* leaves.^41^ The slight variation in peak position among studies can be attributed to differences in extract composition, synthesis conditions, and particle size distribution. Overall, this agreement supports the successful synthesis and confirms the expected optical behavior of ZnONPs.

**Fig. 1:**
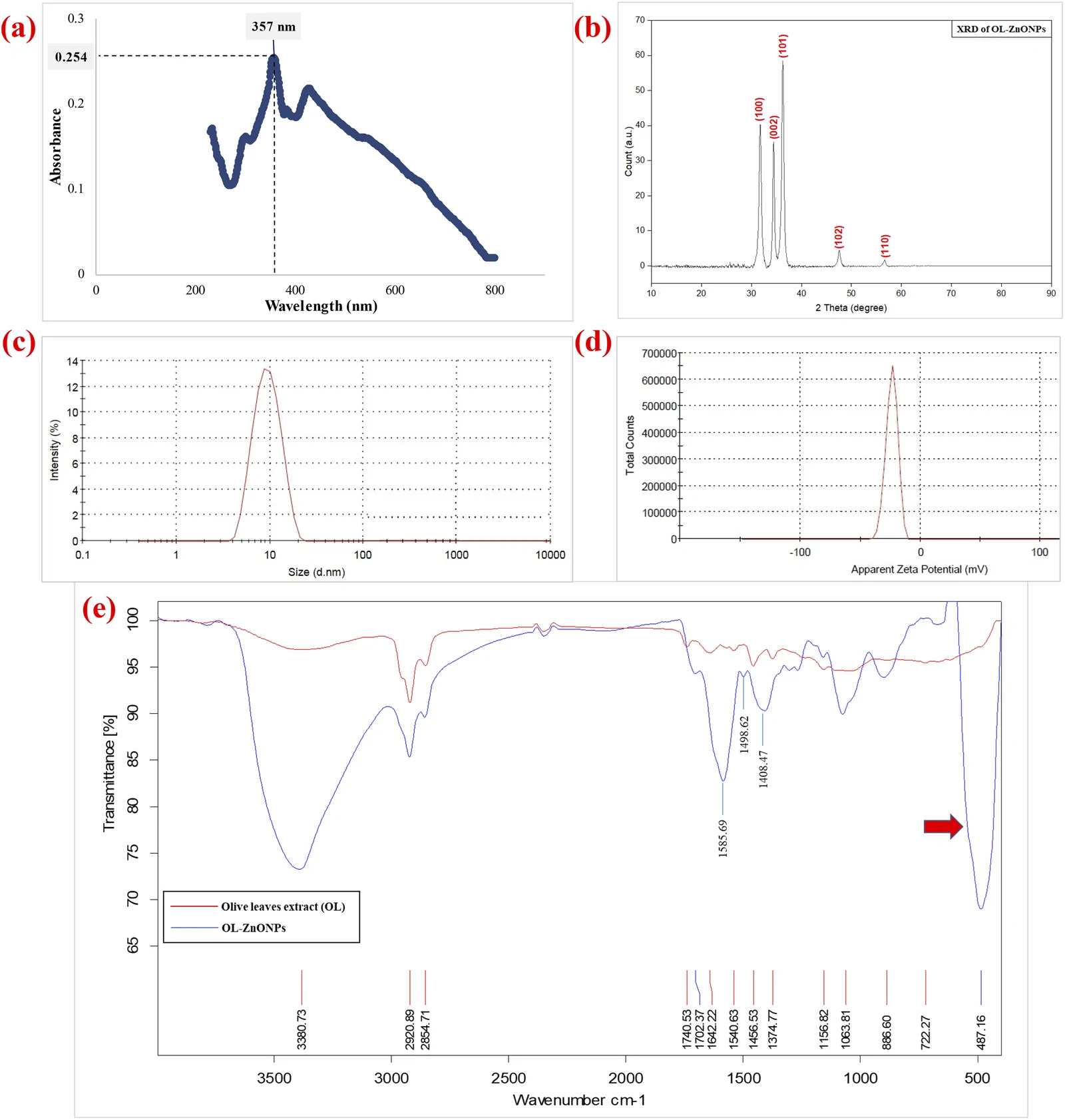
Characterization of zinc oxide nanoparticles synthesized using olive leaf extract (OL-ZnONPs): (a) UV-Vis absorption spectrum showing a distinct peak at 357 nm, confirmed nanoparticle formation; (b) X-ray diffraction (XRD) pattern of the dried OL-ZnONPs powder, revealing characteristic crystalline planes indexed to the hexagonal wurtzite structure of ZnO; (c) dynamic light scattering (DLS) profile indicating a narrow particle size distribution; (d) zeta potential measurement demonstrating a surface charge of −23.09 mV, indicating moderate colloidal stability; and (e) FTIR spectra of the ethanolic olive leaf extract and OL-ZnONPs, highlighting the presence of a Zn–O stretching vibration between 400–500 cm^−1^ in the synthesized sample.

#### 3.1.2 X-ray diffraction (XRD)

X-ray diffraction (XRD) characterizes the crystalline structure of nanoparticles and estimates the crystallite domain size based on diffraction peak broadening using Scherrer's equation. It reveals the crystal phase and degree of crystallinity. It is a volume-averaged technique and does not necessarily reflect the physical particle size. Its accuracy decreases for amorphous materials or particles smaller than approximately 3 nm or larger than 100 nm.^42^

XRD analysis of the synthesized ZnO nanoparticles revealed distinct diffraction peaks at 2*θ* values of 31.76°, 34.44°, 36.27°, 47.57°, and 56.59°, corresponding to the (100), (002), (101), (102), and (110) crystallographic planes, respectively (Fig. 1b). These reflections were consistent with the standard data provided in ICDD reference cards (PDF No. 05-0664 and PDF No. 01-079-0207), confirming phase purity and structural alignment. The diffraction pattern indicated the formation of a hexagonal wurtzite phase (space group *P*6_3_*mc*), characteristic of the zincite form of ZnO, in agreement with previous reports.^43^

Interplanar spacings (*d*-values) were calculated using Bragg's law:*d* = *λ*/2 sin(*θ*)where *d* is the interplanar spacing (Å), *λ* is the wavelength of Cu Kα radiation (1.5406 Å), and *θ* is the Bragg angle (half of the 2*θ* value). The obtained *d*-spacing values for the OL-ZnONPs closely matched reference values (Table 3), with deviations below 0.01 Å, confirming phase purity and structural integrity. This suggests that Zn^2+^ ions were successfully integrated into the wurtzite lattice without significant distortion, which is critical for maintaining nanoparticle functionality.

**Table 3:** Crystallographic parameters and structural features of ZnO nanoparticle synthesized using olive leaf extract^a^

No.	Indices	2*θ* (degree)	FWHM (degree)	Crystallite size *D* (nm)	Area (a.u.)	*d*-spacing of OL-ZnONPs (Å)	*d*-spacing of ref. code 05-0664 (Å)	*d*-spacing of ref. code 01-079-0207 (Å)
1	100	31.76314	0.59434	13.89703129	27.79595	2.8149	2.81600	2.82049
2	002	34.44844	0.43906	18.94340712	18.72413	2.6014	2.60200	2.60626
3	101	36.27398	0.56886	14.69542233	37.77798	2.4745	2.47600	2.48062
4	102	47.57461	0.60423	14.36839877	3.65747	1.9098	1.91100	1.91416
5	110	56.59842	0.58397	15.45051942	1.58129	1.6248	1.62600	1.62841

a FWHM: full width at half maximum; OL-ZnONPs: olive leaves extract-mediated zinc oxide nanoparticles.

Average crystallite size (*D*) was calculated using Scherrer's equation:*D* = *Kλ*/*β* cos *θ*where *D* is the average crystallite size (coherent crystalline regions; nm) based on the diffraction peak width, *K* is the shape factor (0.89), *λ* is the wavelength of Cu Kα radiation (0.15406 nm), *β* is the full width at half maximum (FWHM, in radians) of the peak at 2*θ* in the pattern, and *θ* is the Bragg angle (radians). Analysis of the five diffraction peaks (Table 3) produced an average crystallite size of 15.47 nm, with a crystallinity of 82.80%, confirming nanoscale synthesis and a well-ordered crystal structure.

These results align with previously reported ZnONPs synthesized using aqueous olive leaf extracts, which exhibited crystallite sizes of 17.41 nm and 19.22 nm,^41,43^ demonstrating the reproducibility of this green synthesis approach. The sharp and narrow peaks observed in the XRD pattern confirmed the fine crystalline structure of the biosynthesized ZnO nanoparticles.^44^ Moreover, the small crystallite size suggests a high surface-to-volume ratio, enhancing physicochemical reactivity and potentially improving biological performance, as previously described.^45^

#### 3.1.3 Dynamic light scattering and zeta potential

Dynamic light scattering (DLS) was employed to assess the hydrodynamic diameter and size distribution of OL-ZnONPs. The analysis revealed an average particle size of 10.81 nm, with a polydispersity index (PDI) of 0.395, indicating a relatively narrow size distribution and nearly monodisperse behavior (Fig. 1c). These values reflect the particle size together with the surrounding solvent, including the solvation layer and possible agglomerates, typically yielding larger sizes compared to direct imaging techniques such as TEM, which measure the actual particle size.^44^

Zeta potential analysis, performed *via* electrophoretic light scattering (ELS), revealed a peak value of −23.7 mV (Fig. 1d). This suggests that negatively charged groups constituted the majority of the capping biomolecules on the biosynthesized ZnONPs. The negative surface charge induces strong electrostatic repulsion between particles, thereby maintaining high colloidal stability in suspension.^44^

#### 3.1.4 Fourier transform infrared spectroscopy (FTIR)

FTIR spectra (Fig. 1e) of both OL extract powder and the OL-ZnONPs, recorded in the range of 4000–400 cm^−1^, displayed characteristic absorption bands, confirming the involvement of plant-derived phytochemicals in nanoparticle formation. A broad absorption band around 3380 cm^−1^, assigned to O–H stretching of hydroxyl and phenolic groups, showed a noticeable change in intensity after nanoparticle synthesis, indicating the participation of these functional groups in the reduction and stabilization process.

In addition, C–H stretching vibrations at 2920 and 2854 cm^−1^ were present in both spectra, suggesting that alkyl groups from the OL extract remained adsorbed on the nanoparticle surface. In the carbonyl (C=O) region (1740–1642 cm^−1^), absorption bands associated with esters, aldehydes, ketones, or amide I groups exhibited slight shifts following nanoparticle formation, indicating the involvement of carbonyl-containing biomolecules in Zn^2+^ coordination. Additional spectral changes were observed in the range of 1585–1408 cm^−1^, corresponding to aromatic C=C stretching, amide II vibrations, and CH_2_/CH_3_ bending,^41^ and in the region of 1156–1063 cm^−1^, assigned to C–O/C–O–C/C–N stretching vibrations of alcohols, esters, and polysaccharides. These features highlight the role of flavonoids, proteins, and polysaccharides in capping and stabilizing ZnO nanoparticles.

The observed shifts and variations in band intensities between the OL extract and OL-ZnONPs spectra indicate strong interactions between bioactive compounds and zinc ions during the biosynthesis. Significantly, the OL-ZnONPs spectrum exhibited a new absorption band at 487 cm^−1^, indicative of Zn–O stretching vibrations and confirming successful nanoparticle formation. This band, clearly shown in Fig. 1e, is in agreement with earlier reports that observed a similar band near 480 cm^−1^ during ZnONP biosynthesis using aqueous OL extract.^41^

#### 3.1.5 Transmission electron microscopy (TEM) and selected area electron diffraction (SAE.D)

Transmission electron microscopy (TEM) was used to analyze the morphological characteristics, particle size, and distribution of OL-ZnONPs (Fig. 2a and b). The nanoparticles were predominantly spherical to quasi-spherical, with occasional irregularly shaped nanocrystals, and tended to form closely packed, grape-like aggregates. Although such agglomeration may reduce effective surface area and influence biological activity, it can prolong circulation time and delay systemic clearance, thereby enhancing nanoparticle retention and bioavailability.^46^

**Fig. 2:**
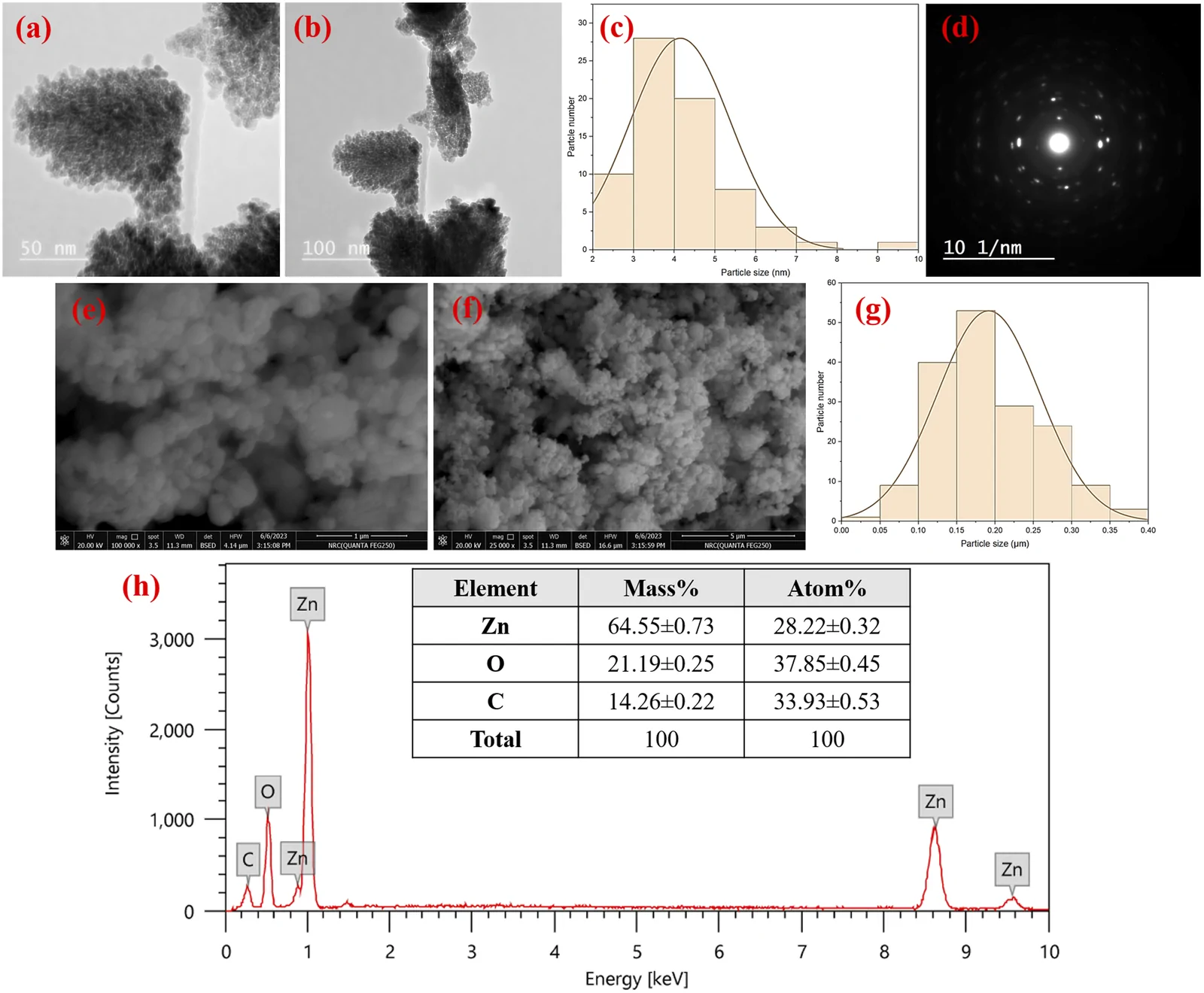
Characterization of synthesized ZnO nanoparticles (OL-ZnONPs): (a & b) TEM micrographs (scale bar: 50 and 100 nm, respectively); (c) histogram of particle size (calibrated using the 100 nm scale bar); (d) SAED pattern; (e & f) SEM micrographs (scale bar: 1 and 5 µm, respectively); (g) histogram of particle size (calibrated using the 5 µm scale bar); and (h) energy dispersive X-ray (EDX) spectrum of OL-ZnONPs.

Representative TEM micrographs at two magnifications are shown in Fig. 2a and b. Particle diameters ranged from 2.37 to 9.18 nm, with an average size of 4.15 ± 1.23 nm (SD, *n* = 71), calculated from the high-magnification micrograph (calibrated using the 100 nm scale bar). The corresponding particle size distribution is illustrated in a histogram (Fig. 2c).

In addition, selected area electron diffraction (SAE.D) analysis further verified the crystalline nature of OL-ZnONPs (Fig. 2d). Well-defined diffraction rings and discrete spots were observed, consistent with the hexagonal wurtzite phase identified by XRD analysis. These findings corroborate the crystallinity and phase purity of biosynthesized OL-ZnONPs.

Finally, TEM provides direct visualization of individual dry particles, reflecting the core size without the surrounding medium. This explains why the TEM-derived mean diameter is smaller than the value obtained by dynamic light scattering (DLS, 10.81 nm), which represents the hydrodynamic diameter including the solvation layer and the phytochemical coating.

The discrepancy between the larger crystallite size obtained by XRD (16.63 nm) and the TEM-derived particle size arises from the fundamental differences in measurement principles between the two techniques. TEM directly measures the physical dimensions of individual nanoparticles, providing a number-averaged particle size. In contrast, XRD *via* the Scherrer equation measures the average size of coherently diffracting crystalline domains (crystallites) across the entire sample volume, yielding a volume-averaged crystallite size. Therefore, the two techniques do not measure the same physical quantity.^47^

The larger crystallite size obtained from XRD can be attributed to the presence of polycrystalline agglomerates, where the XRD signal is dominated by larger coherently diffracting domains that may encompass several smaller primary particles observed individually by TEM. Additionally, the Scherrer equation does not account for microstrain, which can contribute to peak broadening and lead to an underestimation of the crystallite size. The Scherrer method is generally applicable to crystallite sizes ranging from approximately 1 to 100 nm, with optimal accuracy within the range of approximately 3 to 80 nm.^42^ Particle sizes smaller than 3 nm typically result in excessively broad diffraction peaks, as a particle may contain smaller domains where all moments are aligned in the same direction, even if the particle itself is a single domain.^47^ Conversely, particle sizes larger than 100 nm are heavily influenced by instrumental resolution, making precise evaluation difficult; for particles exceeding 50 nm, multiple crystal boundaries may exist on the surface, and XRD cannot distinguish between them.^42^ Therefore, the observed difference between the XRD and TEM values is not only reasonable but expected. The particle size data obtained by TEM provide a more accurate representation of the actual physical particle size.

#### 3.1.6 Scanning electron microscopy (SE.M) and energy dispersive X-ray spectroscopy (EDX)

SE.M micrographs revealed that OL-ZnONPs consisted primarily of spherical to quasi-spherical particles assembled into irregular, densely packed aggregates visible at different magnifications (Fig. 2e and f). SEM analysis corroborated the morphological features observed in TEM findings. Aggregation may result from the high surface energy of ZnONPs,^48^ adsorption of OL-derived biomolecules acting as capping and stabilizing agents, and interparticle interactions driven by van der Waals forces, electrostatic effects, and hydration.^43^

The particle size ranged from 0.047 to 0.388 µm, with a mean diameter of 0.192 ± 0.067 µm (SD, *n* = 168), calibrated using the 5 µm scale bar. These values reflect the size of secondary agglomerates formed during drying rather than individual crystallites, explaining their larger magnitude compared to TEM and DLS measurements (Fig. 2g).

The elemental composition and purity of OL-ZnONPs were further evaluated using energy dispersive X-ray spectroscopy (EDX). As shown in Fig. 2h, the spectrum revealed zinc, oxygen, and carbon, with weight percentages of 64.55%, 21.19%, and 14.26%, respectively. After excluding carbon as a non-structural component, the elemental composition of ZnO nanoparticles was calculated to be 75.29% Zn and 24.71% O, as processed previously in,^49^ which was consistent with previous studies documenting 76.53% Zn and 23.47% O.^41^ These findings confirm the successful formation of ZnO nanoparticles and support the reproducibility of the green synthesis strategy.

### 3.2 Targeted LC-ESI-MS/MS approach in MRM mode

#### 3.2.1 LC-ESI-MS/MS analysis in MRM mode

A targeted LC-ESI-MS/MS analysis in MRM mode was performed to profile the phenolic constituents of the 70% ethanolic olive leaf (OL) extract, both qualitatively and quantitatively. Compound identification was achieved by comparing their spectral features and retention times with authentic reference standards. Twenty-one phenolic acids and flavonoids were selected as standards based on the previously described methodology section. Although secoiridoids are major olive leaf constituents, the focus on phenolic acids and flavonoids was justified by their direct relevance to nanoparticle biosynthesis. Identification was achieved by comparing retention times and specific precursor/product ion transitions (Q1/Q3) with authentic standards (Table 4 and Fig. 3a).

**Table 4:** Quantitative and validation parameters for phenolic acids and flavonoids detected in the 70% ethanolic olive leaf extract using LC-ESI-MS/MS in MRM mode^a^

No.	Compound name	Expected Rt	Experimental Rt	Conc. (µg g^−1^)	*R* ^2^	LOD (µg g^−1^)	LOQ (µg g^−1^)
1	Gallic acid	1.67	1.81	5.52 ± 0.05	0.9962	0.0866	0.2623
2	Caffeic acid	6.19	6.19	10.43 ± 0.1	0.9977	0.0674	0.2043
3	Rutin	9.53	9.53	354.15 ± 0.1	0.9970	0.0771	0.2337
4	Coumaric acid	8.13	8.13	21.36 ± 0.07	0.9988	0.0496	0.1504
5	Vanillin	7.48	N/A	ND	0.9990	0.0484	0.1468
6	Naringenin	20.98	N/A	ND	0.9979	0.0687	0.2082
7	Quercetin	18.78	18.78	45.75 ± 0.13	0.9972	0.0746	0.2261
8	Ellagic acid	8.97	N/A	ND	0.9984	0.0559	0.1695
9	3,4-Dihydroxybenzoic acid	3.13	3.4	78.21 ± 0.07	0.9988	0.0489	0.1481
10	Hesperetin	22.62	N/A	ND	0.9983	0.0579	0.1754
11	Cinnamic acid	18.16	N/A	ND	0.9989	0.0490	0.1486
12	Methyl gallate	5.04	N/A	ND	0.9981	0.0614	0.1860
13	Kaempferol	22.64	22.64	1.95 ± 0.03	0.9983	0.0582	0.1762
14	Ferulic acid	8.89	9.37	13.11 ± 0.07	0.9961	0.0884	0.2679
15	Syringic acid	6.58	6.58	4.11 ± 0.1	0.9986	0.0520	0.1577
16	Apigenin	21.47	22.01	4.67 ± 0.08	0.9984	0.0608	0.1844
17	Catechin	5.05	N/A	ND	0.9991	0.0446	0.1353
18	Daidzein	16.22	N/A	ND	0.9952	0.0978	0.2964
19	Chlorogenic acid	5.42	5.42	40.49 ± 0.08	0.9984	0.0561	0.1702
20	Resveratrol	14.27	N/A	ND	0.9968	0.0796	0.2412
21	Rosmarinic acid	13.42	N/A	ND	0.9975	0.0697	0.2111

a ND, not detected; N/A, not applicable; *R*^2^, coefficient of determination.; LOD, limit of detection.; LOQ, limit of quantification.

**Fig. 3:**
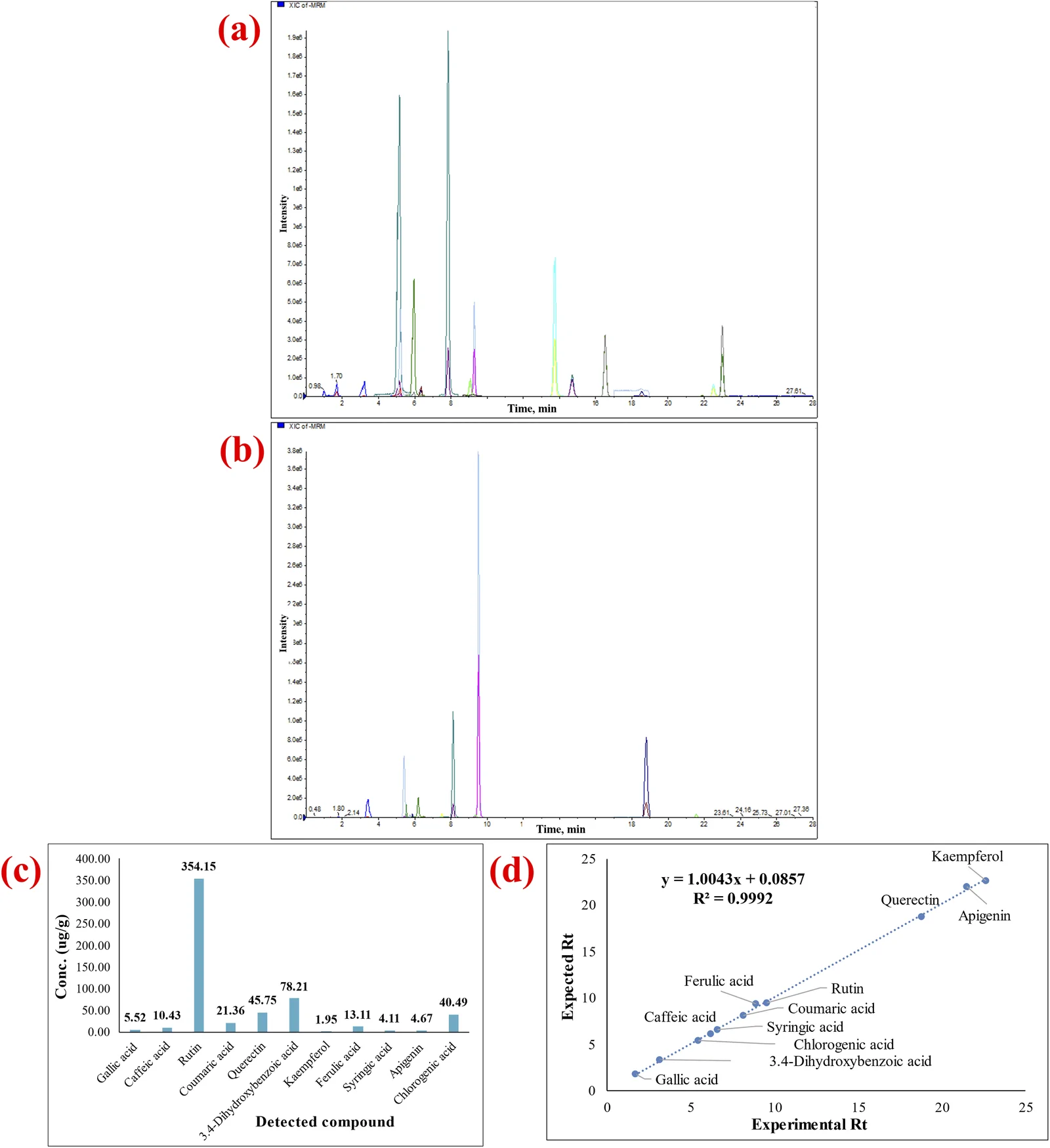
LC-ESI-MS/MS chromatograms acquired in MRM mode under negative ionization for selected phenolic acids and flavonoids. (a) chromatogram of 11 standard compounds; (b) corresponding chromatogram of the 70% ethanolic olive leaf extract; (c) bar chart illustrating the concentration (µg g^−1^) of the detected phenolic compounds, with rutin exhibiting the highest concentration; (d) Scatter plot comparing the expected and experimental retention times (Rt) for the identified compounds, demonstrating strong linearity (*R*^2^ = 0.9992) and confirming the reliability and accuracy of compound identification under the applied chromatographic conditions.

Eleven compounds were detected (Fig. 3b), with concentrations ranging from 1.95 to 354.15 µg g^−1^. Rutin (354.15 µg g^−1^), 3,4-dihydroxybenzoic acid (78.21 µg g^−1^), and quercetin (45.75 µg g^−1^) were the most abundant (Fig. 3c). Chlorogenic acid, coumaric acid, ferulic acid, and caffeic acid were present at moderate levels, whereas gallic acid, apigenin, syringic acid, and kaempferol were detected at lower concentrations. These findings are consistent with previous LC-MS/MS-MRM profiling of OL extracts prepared under different extraction conditions, which similarly identified rutin and quercetin as dominant phenolics in hot water extracts,^31^ while rutin and 3,4-DHBA were most abundant in 80% methanolic extracts.^30^ These results support the rationale for focusing on phenolic acids and flavonoids in the present study. Overall, the compositional profile highlights olive leaves as a rich source of structurally diverse phenolic compounds, supporting their suitability as bioactive matrices for green nanoparticle synthesis and potential nutraceutical applications.

#### 3.2.2 Retention time consistency and system robustness

To assess chromatographic reproducibility, a scatter plot was generated by comparing expected and experimental retention times (Fig. 3d). Linear regression analysis produced the equation *y* = 1.0043*x* − 0.0857, with coefficient of determination (*R*^2^) equal to 0.9992, confirming retention time consistency across analytes and the robustness of the LC-ESI-MS/MS system for reliable compound identification.

#### 3.2.3 Sensitivity evaluation and detection limits

Method sensitivity was evaluated by calculating LOD and LOQ for each analyte using calibration curves constructed from authentic standard solutions. These values were derived from the ratio of the residual standard deviation to the slope of the calibration curve. All compounds exhibited strong linearity (*R*^2^ > 0.995; Fig. 4 and S2). The calculated LOD values ranged from 0.0446 to 0.0978 µg g^−1^, while LOQs ranged from 0.1353 to 0.2964 µg g^−1^ (Table 4). Compounds such as vanillin, ellagic acid, and catechin were not detected in the OL extract, despite having low LOD values, indicating their absence or presence below the quantifiable range of the method.

**Fig. 4:**
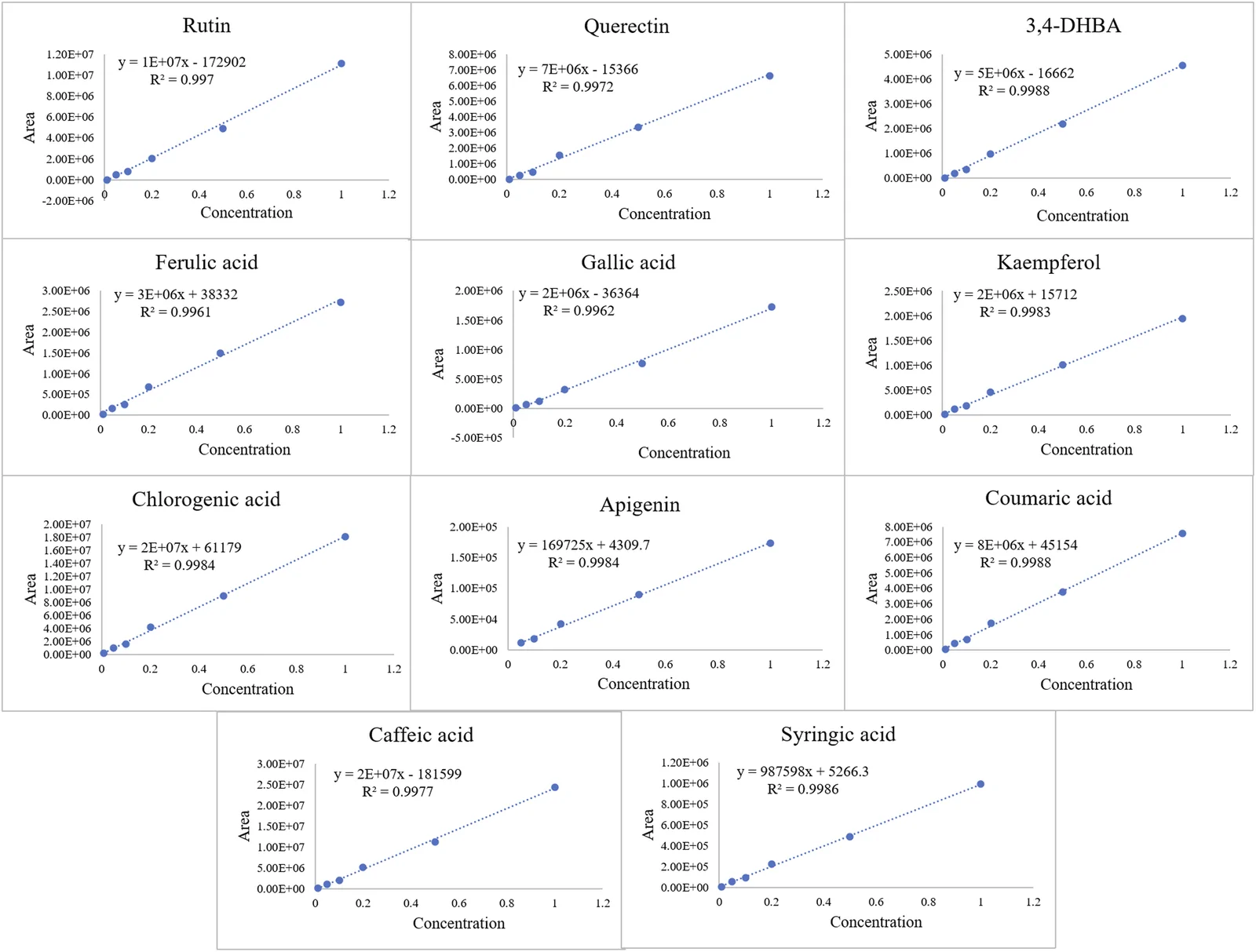
Calibration curves of 11 phenolic standards quantified *via* LC-ESI-MS/MS in negative ionization MRM mode. Each curve represents the linear relationship between concentration and peak area, with corresponding regression equations and *R*^2^ values, demonstrating high analytical accuracy and reproducibility.

### 3.3 Anti-cancer activity *in vitro*

#### 3.3.1 Cytotoxicity assessment on normal cells (HSF)

The cytotoxicity of OL-ZnONPs, olive leaf extract, and the reference drug 5-fluorouracil (5-FU) was assessed in human skin fibroblast (HSF) cells using the MTT assay at concentrations ranging from 1.25 to 40 µg mL^−1^ for 48 h (Table S1). All treatments induced a concentration-dependent reduction in cell viability, as confirmed by statistical analysis (Fig. S3a).

OL-ZnONPs exerted the strongest effect in HSF cells, with the lowest EC_50_ value among the tested treatments (1.816 ± 0.8244 µg mL^−1^), compared with 5-FU (3.19 ± 0.97 µg mL^−1^) and the crude extract (20.02 ± 1.65 µg mL^−1^) (Table 5). Similar observations have been reported for *Cassia fistula*-derived ZnO nanoparticles, which showed marked cytotoxicity in NIH-3T3 fibroblasts compared to commercial ZnO samples.^50^ These findings indicate that biosynthesized ZnONPs, while showing anticancer potential, may also exert measurable cytotoxic effects on normal cell. The cytotoxic response to ZnONPs may be influenced by factors such as particle size, surface characteristics, and the phytochemical composition associated with nanoparticle synthesis.^51,52^ Therefore, the observed cytotoxicity of OL-ZnONPs in HSF cells highlights the importance of evaluating both anticancer activity and normal-cell toxicity when assessing their therapeutic potential. Further *in vivo* studies are required to establish their biosafety and therapeutic efficacy.

**Table 5:** EC_50_, IC_50_, and selectivity indexes (SI) values of the tested samples on normal cells and cancer cell lines^a^

Cell type	Cytotoxicity parameters	Cell line	Tested samples (µg mL^−1^)		
			OL-ZnONPs	OL extract	5-FU
Normal cell	EC_50_	HSF	1.816 ± 0.8244	20.02 ± 1.6497	3.189 ± 0.9677
Cancer cell lines	IC_50_	HepG-2	0.344 ± 0.02	15.00 ± 0.645	3.383 ± 1.349
		MDA-MB-231	0.4199 ± 0.031	11.30 ± 0.834	3.756 ± 1.814
		HCT-116	1.008 ± 0.08	16.78 ± 1.063	3.592 ± 1.425
	SI	HepG-2	5.279	1.33	0.943
		MDA-MB-231	4.325	1.77	0.849
		HCT-116	1.802	1.19	0.888

a C_50_, half-maximal effective concentration; IC_50_, half-maximal inhibitory concentration; SI, selectivity index.

#### 3.3.2 Anticancer activity on cancer cell lines

This study provides a comparative analysis of olive-leaf-mediated ZnONPs against liver, breast, and colon carcinoma cell lines, with a quantified selectivity index relative to the reference drug (5-FU) and olive leaf extract (Fig. 5). The antiproliferative effects of OL-ZnONPs, OL extract, and 5-FU were examined in HepG-2 (hepatocellular carcinoma), MDA-MB-231 (breast carcinoma), and HCT-116 (colon carcinoma) cell lines (Fig. S3 and Table 5).

**Fig. 5:**
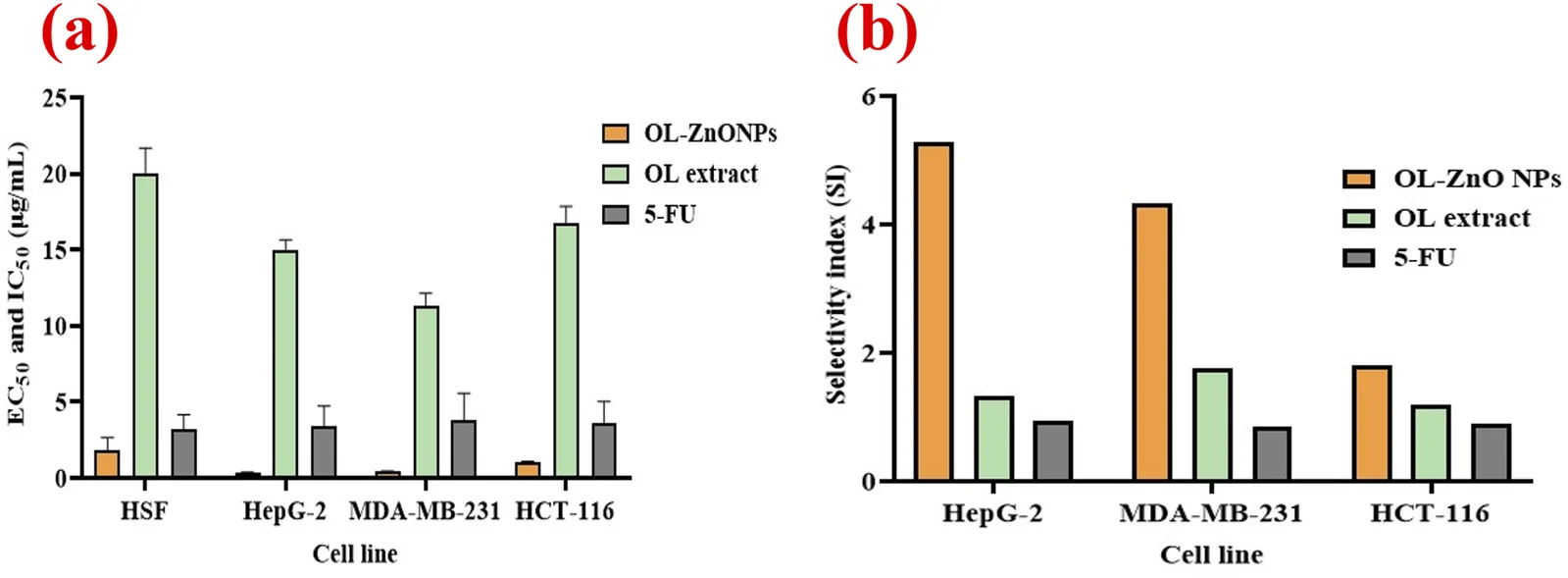
(a) Cytotoxicity assessment of OL-ZnONPs, OL extract, and 5-FU against normal human skin fibroblasts (HSF) and cancer cell lines (HepG-2, MDA-MB-231, HCT-116) showing EC_50_ and IC_50_ values; (b) selectivity index (SI) analysis demonstrating the highest selectivity of OL-ZnONPs toward HepG-2 cells, compared to OL extract and 5-FU.

OL-ZnONPs demonstrated potent cytotoxic activity across all tested cancer cells, with HepG-2 cells showing the greatest sensitivity (IC_50_ = 0.3440 µg mL^−1^; SI = 5.279). MDA-MB-231 cells also showed high sensitivity to OL-ZnONPs, with an IC_50_ of 0.4199 ± 0.031 µg mL^−1^ and an SI of 4.325, whereas HCT-116 cells exhibited moderate sensitivity to OL-ZnONPs, with an IC_50_ of 1.008 ± 0.08 µg mL^−1^ and an SI of 1.802. Thus, HepG-2 showed the lowest IC_50_ and highest SI among the three tested cancer cell lines (Fig. 6a). MDA-MB-231 cells also responded strongly, consistent with earlier reports of dose-dependent viability reduction in this model, where IC_50_ values shifted from 75 µg mL^−1^ at 24 h to 50 µg mL^−1^ at 48 h using the MTT assay.^19^ The OL extract displayed measurable antiproliferative activity, although it was less potent than OL-ZnONPs. In contrast, 5-FU exhibited cytotoxicity toward both normal and cancer cells, with SI values below 1.0 (0.943, 0.849, and 0.888 for HepG-2, MDA-MB-231, and HCT-116, respectively), indicating limited selectivity between malignant and normal cells under the tested conditions. These findings indicate that OL-ZnONPs showed more favorable *in vitro* selectivity profile than the tested reference treatments, particularly toward HepG-2 cells.

**Fig. 6:**
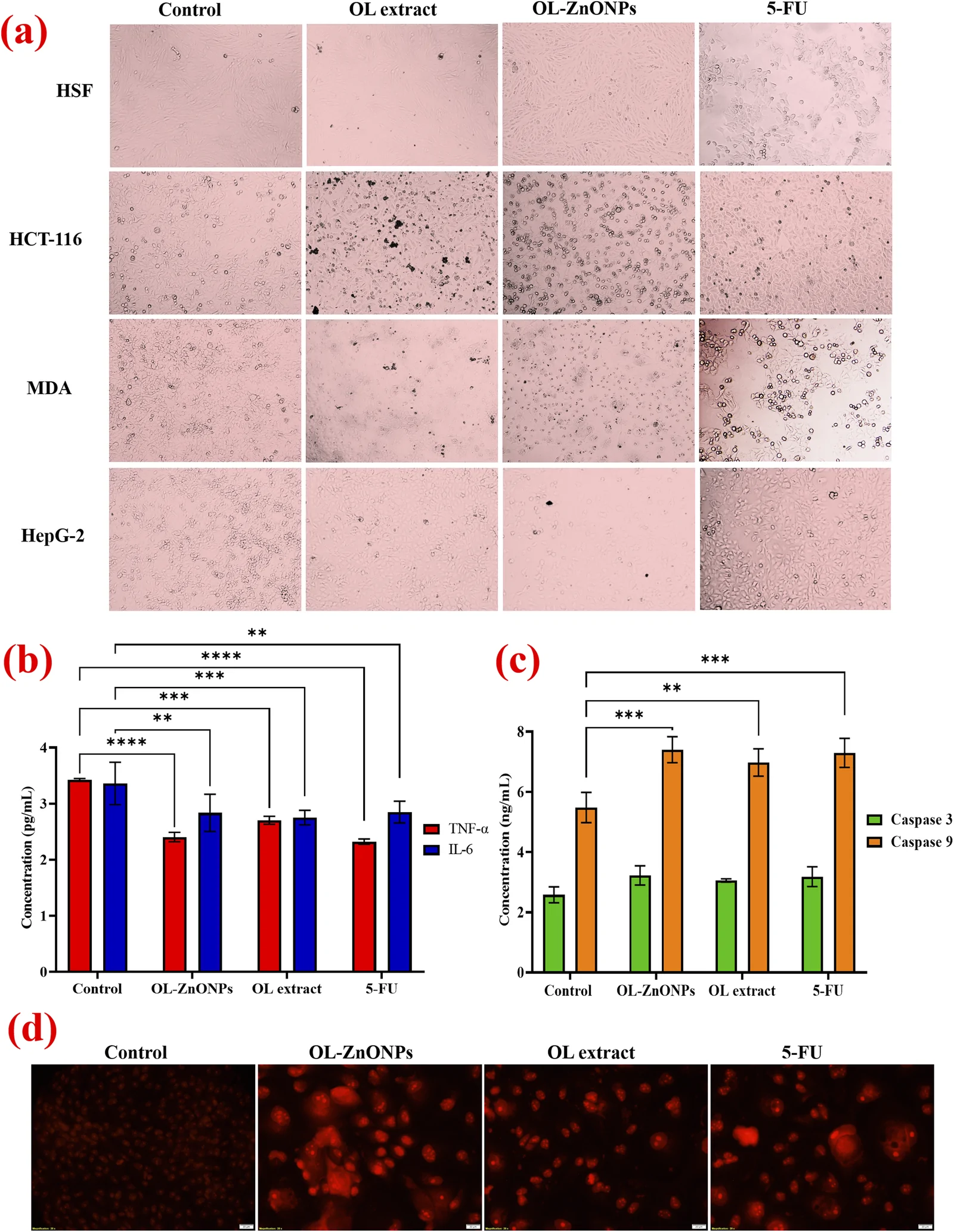
(a) Bright-field microscopy images of normal and cancer cell lines treated with OL-ZnONPs, OL extract, and 5-FU at concentrations corresponding to the EC_50_ for HSF cells and IC_50_ values for cancer cell lines. (b and c) Quantitative analysis of pro-inflammatory markers (TNF-α, IL-6) and apoptosis-related markers (caspase-3, caspase-9), respectively, in HepG-2 cells following treatment with OL-ZnONPs, OL extract, and 5-FU. Data are presented as mean ± SD (*n* = 3). Statistical significance is indicated as follows: ****p < 0.0001; ***p < 0.001; **p < 0.01. (d) Fluorescence micrographs of HepG-2 cells stained with propidium iodide following treatment with OL-ZnONPs, OL extract, and 5-FU.

The SI values of 5.279 for HepG-2 and 4.325 for MDA-MB-231 exceeded the SI threshold of 2.0 reported as indicative of promising selectivity for anticancer agents.^53^ Although these results are promising, further dose optimization and surface functionalization may be investigated in future studies to further characterize therapeutic window and improve the selectivity profile and biocompatibility of OL-ZnONPs.

The anticancer potency of the OL-ZnONPs was higher under the reported experimental conditions than that of several previously reported green-synthesized ZnO formulations. For instance, biogenic ZnO nanoparticles mediated by *Celosia argentea* and *Echinops spinosus* root extracts required much higher concentrations to reach 50% inhibition in the HepG-2 cell (49.45 µg mL^−1^ and 19.94 µg mL^−1^, respectively).^10,15^ Similarly, formulations synthesized from *Elaeagnus angustifolia* (21.7 µg mL^−1^) and *Moringa oleifera* (21.0–419.0 µg mL^−1^) showed higher reported IC_50_ values in hepatocellular carcinoma models.^54,55^ In breast cancer cells, OL-ZnONPs achieved an IC_50_ of 0.4199 µg mL^−1^ against MDA-MB-231, which was lower than the reported IC_50_ values of ZnO nanoparticles derived from *Brassica napus* pollen (1.0 µg mL^−1^) or *Paulownia tomentosa* (45.2 µg mL^−1^) in MCF-7.^56,57^

The potent activity of OL-ZnONPs may reflect the combined contribution of the ZnO nanostructure and the olive-leaf-derived bioactive compounds associated with the nanoparticle surface. However, the relative contribution of each component, as well as any potential synergistic interaction between them, cannot be determined in the absence of a size-matched bare ZnO control.

#### 3.3.3 Quantification of pro-inflammatory and apoptosis-related markers

The effects of OL-ZnONPs, OL extract, and 5-FU on apoptosis-related markers (caspase-3 and caspase-9) and inflammation-related markers (TNF-α and IL-6) in HepG-2 cells are presented in Fig. 6b and c. Treatment with OL-ZnONPs markedly reduced the levels of the pro-inflammatory cytokines IL-6 and TNF-α relative to control cells, suggesting attenuation of the inflammatory response. In parallel, OL-ZnONPs significantly increased the intracellular levels of total caspase-3/9 compared with untreated control cells, supporting the involvement of apoptosis-related pathways.

These findings are consistent with previous studies reporting the apoptotic and anti-inflammatory effects of ZnONPs. For instance, ZnONPs have been shown to induce apoptosis through caspase-related pathways.^11^ Similarly, suppression of pro-inflammatory cytokines such as IL-6 and TNF-α has been reported in association with the anticancer effects of ZnONPs.^9^ Collectively, these findings show that OL-ZnONPs treatment was associated with reduced IL-6 and TNF-α levels and increased intracellular levels of total caspase-3/9 in HepG-2 cells, indicating alterations in inflammation- and apoptosis-related markers following treatment.

#### 3.3.4 Apoptotic evaluation by nuclear staining

Cancer cells treated with OL-ZnONPs, OL extract, or 5-FU at their respective IC_50_ levels showed morphological changes consistent with apoptosis, including cell shrinkage, nuclear condensation, and membrane blebbing. PI nuclear staining (Fig. 6d) further revealed nuclear condensation and fragmentation in OL-ZnONPs-treated HepG-2 cells, consistent with their greater sensitivity. In contrast, control cells displayed minimal PI uptake and largely preserved nuclear morphology.

These observations align with earlier studies reporting apoptosis-related morphological changes in MDA-MB-231 cells treated with OL-ZnONPs, in which dose-dependent viability reduction was accompanied by cell shrinkage, aggregation, deformation, nuclear condensation, and a reduced proliferation rate.^19^ The observed nuclear alterations, together with increased intracellular levels of total caspase-3/9, support the involvement of apoptosis in the cytotoxic response to OL-ZnONPs.

#### 3.3.5 Migration inhibition assay (scratch assay)

Cell migration plays a central role in cancer progression and metastatic spread; therefore, the effects of OL-ZnONPs, OL extract, and 5-FU on HepG-2 cells were assessed using a scratch assay. Representative phase-contrast images illustrating scratch closure at 0, 24, and 48 h are shown in Fig. 7a. OL-ZnONPs-treated cells showed a visible delay in scratch closure compared with untreated controls. Quantitative analysis confirmed a significant reduction in both wound closure percentage and average migration speed after 24 and 48 h in OL-ZnONPs-treated cells (Fig. 7b and c).

**Fig. 7:**
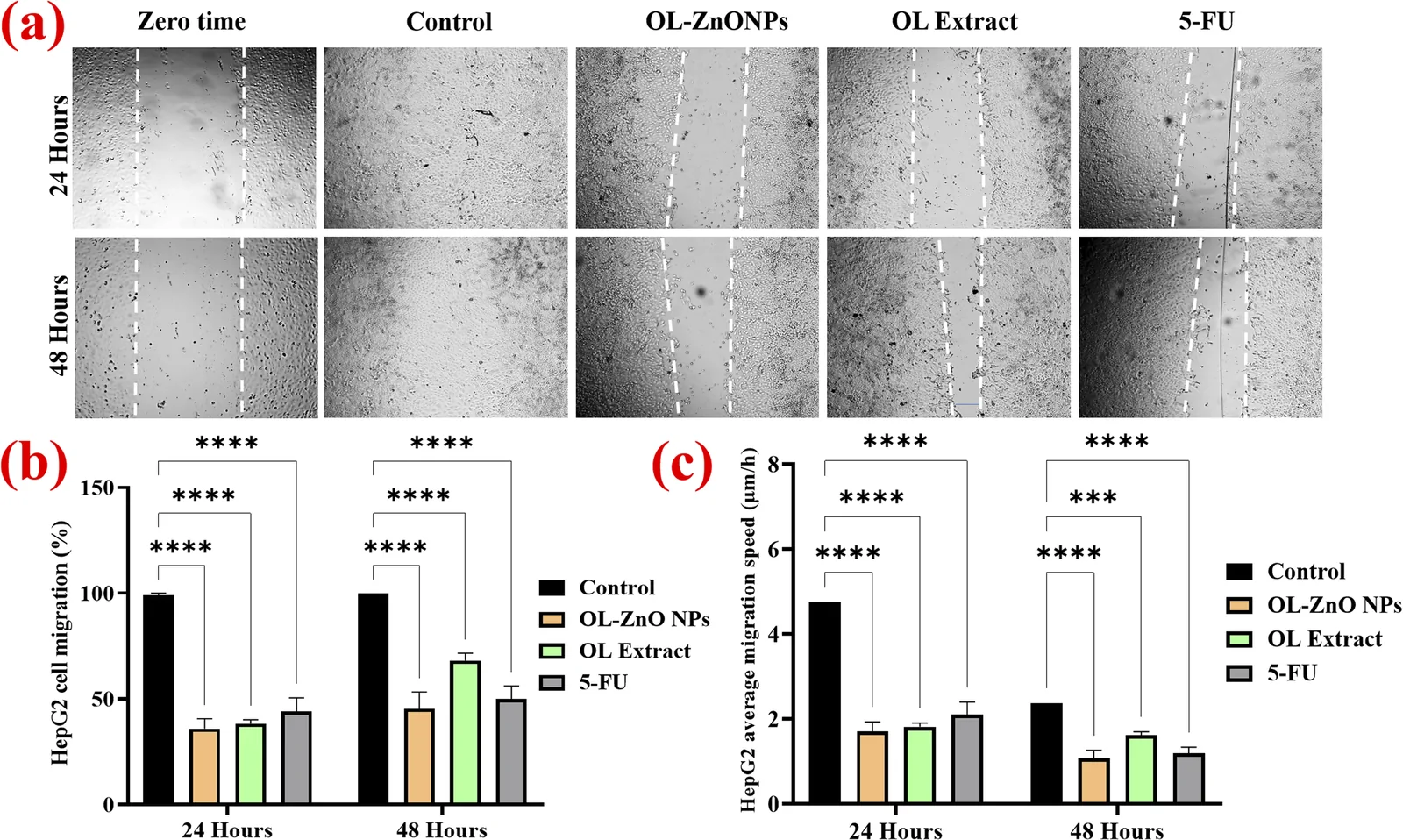
(a) Scratch assay demonstrating the effect of OL-ZnONPs, OL extract, and 5-FU on HepG-2 cell migration after 24 and 48 h of treatment. (b) Quantitative analysis of cell migration percentages. (c) Quantitative assessment of average cell migration speed (µm h^−1^). Data are presented as mean ± SD (*n* = 3). Statistical significance is shown as follows: ****p < 0.0001; ***p < 0.001.

The reduced scratch closure indicates impaired HepG-2 cell motility under the tested conditions. However, the assay was performed at the respective IC_50_ concentrations, the observed reduction may also be influenced by treatment-related effects on cell viability and proliferation. Therefore, the present findings demonstrate reduced migratory capacity but do not establish a specific molecular mechanism. Previous studies have reported that ZnO nanomaterials can affect cellular migration in a concentration- and cell-context-dependent manner.^58^ Collectively, OL-ZnONPs treatment impaired HepG-2 cell migration and scratch closure alongside cytotoxic and apoptosis-related responses observed in this study. Further mechanistic and *in vivo* studies are required to determine the biological significance of this effect.

### 3.4 Network pharmacology analysis

To support the experimental findings, a network pharmacology approach was applied to explore potential molecular associations related to the anticancer activity of olive leaf extract and green-synthesized ZnO nanoparticles. Eleven phenolic compounds identified by LC-ESI-MS/MS, together with ZnO, were analyzed to predict their associated protein targets and biological pathways in the HCC/HepG-2 experimental context.

#### 3.4.1 Compound–target mapping and refinement of disease-related gene targets

The compound target prediction was performed on 11 phenolic compounds identified by LC-MS/MS in MRM mode (rutin, 3,4-dihydroxybenzoic acid, quercetin, chlorogenic acid, coumaric acid, ferulic acid, and caffeic acid, gallic acid, apigenin, syringic acid, and kaempferol). In addition, ZnO-associated targets were included to evaluate its potential contribution to the biological activity of the green-synthesized ZnONPs. The predicted compound targets were mapped to their corresponding proteins and gene names using the UniProt database.

Disease-related targets were retrieved from multiple databases, as described in the methodology section. To provide disease- (HCC) and cell-model (HepG-2) context for target prioritization, 19 322 HCC-associated genes were overlapped with 17 181 HepG-2-associated genes, yielding 16 756 common genes defined as the HCC-HepG-2 reference gene set (Fig. 8a).

**Fig. 8:**
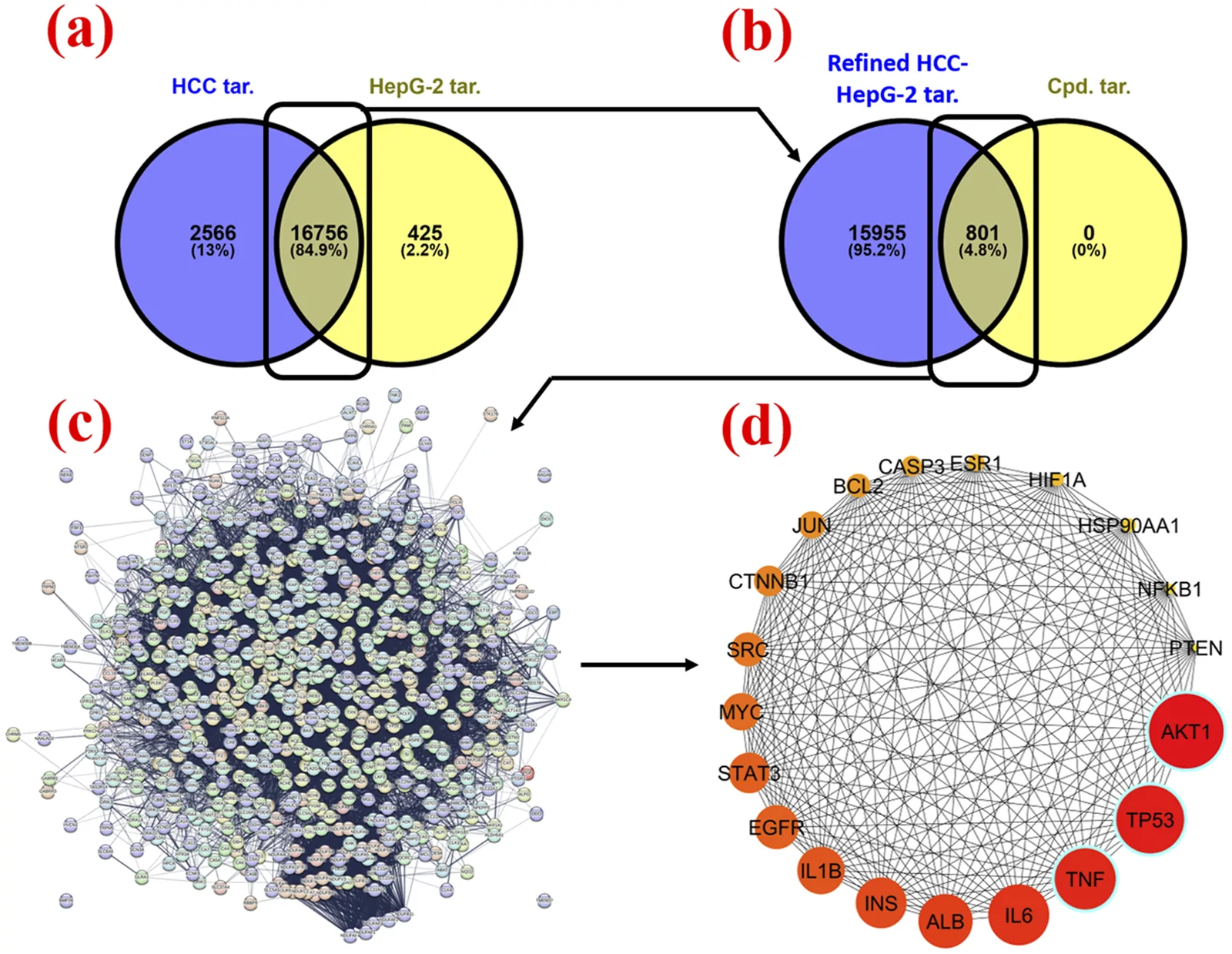
Venn diagram and PPI network analyses of anti-HCC targets. (a) Venn diagram showing the overlap between HCC-associated and HepG-2-associated genes. (b) Venn diagram illustrating the intersection between HCC–HepG-2-associated targets and compound-associated targets. (c) Full protein–protein interaction (PPI) network of the 801 overlapping targets. (d) Filtered PPI network showing the top 20 hub genes, ranked by degree centrality.

This refined gene set was individually intersected with the compound-associated targets. After removal of duplicates, 801 overlapping targets were retained for subsequent computational analyses, including protein–protein interaction, KEGG enrichment, and gene ontology (Fig. 8b). Thus, this reference set represents genes associated with both HCC and HepG-2 rather than an HCC-specific gene set.

#### 3.4.2 PPI network construction and hub gene identification

A protein–protein interaction (PPI) network comprising 801 nodes and 23 785 edges was constructed using the STRING database by uploading the anti-HCC-HepG-2-associated targets (Fig. 8c). In network analysis, a higher node degree reflects a greater functional role of the target. Accordingly, the top 10 hub genes identified by degree centrality were AKT1 (365), TP53 (363), TNF (325), IL6 (315), ALB (305), INS (301), IL1B (298), EGFR (292), STAT3 (292), and MYC (290) (Fig. 8d). These nodes may serve as central anti-HCC targets within the HepG-2 context, originating from the selected OL-ZnONPs components.

Importantly, these computational predictions were concordant with the *in vitro* findings: OL-ZnONPs treatment significantly reduced TNF-α and IL-6 levels while increasing the intracellular levels of total caspase-3 and caspase-9. The concordance between the predicted targets and the observed changes in inflammatory- and apoptosis-related markers provides supportive context for the potential involvement of these biological processes in the cellular response to OL-ZnONPs treatment.

#### 3.4.3 KEGG pathway and GO enrichment analysis

KEGG Pathway enrichment analysis was performed on 801 compound-associated targets, and the top enriched pathways ranked by FDR, as shown in Fig. 9a. Among the highly ranked pathways were pathways in cancer (hsa05200; −log_10_(FDR) = 81.6), followed by chemical carcinogenesis-reactive oxygen species (hsa05208; −log_10_(FDR) = 73.8), PI3K–Akt signaling (hsa04151; −log_10_(FDR) = 43.5), and apoptosis (hsa04210; −log_10_(FDR) = 38.2). These enriched pathways are associated with tumor progression and other diverse processes involved in cancer biology, cellular signaling, and stress responses, as previously described.^59^

**Fig. 9:**
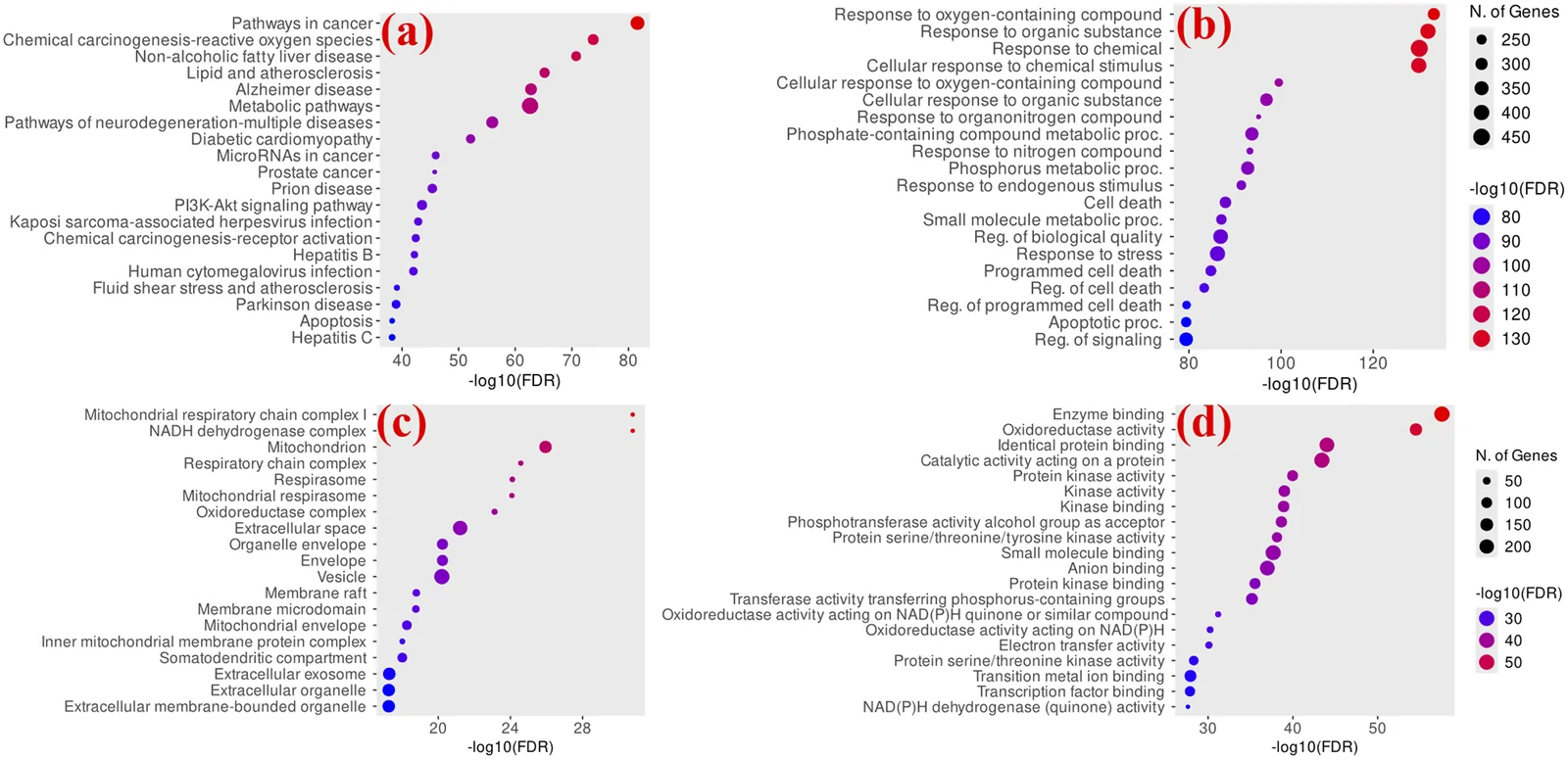
KEGG pathways and Gene Ontology (GO) enrichment analysis of 801 compound-associated targets within the HCC–HepG-2-associated reference gene set. Figures (a–d) display the top 20 enriched pathways and GO terms ranked by −log_10_(FDR). The number of associated genes is represented by dot size, and statistical significance is indicated by color intensity: (a) KEGG pathway enrichment analysis; (b) GO biological process (B.P) enrichment analysis; (c) GO cellular component (CC) enrichment highlighting predominant mitochondrial localization; (d) GO molecular function (MF) enrichment illustrating the major functional activities associated with the target genes.

Of particular relevance, the prominence of apoptosis-related pathways is consistent with the identification of TP53 as a highly connected node (key hub gene) in the PPI network and with the experimental increase in total caspase-3 and caspase-9 levels and the observed nuclear alterations in HepG-2 cells, supporting the involvement of apoptosis-related responses. Other enriched pathways included viral hepatitis, microRNA-related signaling, and non-alcoholic fatty liver disease. These results indicate that the predicted targets are enriched in multiple signaling pathways related to cancer-associated biological processes.

GO biological process (B.P) analysis (Fig. 9b) demonstrated significant enrichment of terms associated with response to oxygen-containing compounds, organic substance, chemical response to chemical stimulus, apoptotic process, cell death, and programmed cell death. These processes are closely associated with cellular stress responses and apoptosis regulation, supporting the possible involvement of programmed cell death-related processes in the cellular response to OL-ZnONPs.^37^ Additional enriched terms included response to organic substances, cellular response to oxygen-containing compounds, and metabolic processes (*e.g.*, small molecule metabolism and phosphate-containing compound metabolism), indicating the broader range of biological processes represented among the predicted targets.

GO cellular component (CC) analysis primarily associated with mitochondria, as well as to respiratory chain complex I, the NADH dehydrogenase complex, the respirasome, and the inner mitochondrial membrane protein complex (Fig. 9c).

GO molecular function (MF) analysis revealed enrichment of terms associated with enzyme binding, oxidoreductase activity, identical protein binding, kinase activity, kinase binding, and transcription factor binding (Fig. 9d).

Taken together, the integrated KEGG and GO enrichment analyses provided a hypothesis-generating view of the biological processes associated with the predicted selected targets of OL-ZnONPs. The enrichment of cancer-related, cellular stress-response, PI3K-Akt, and apoptosis pathways, together with the experimental findings, supports the possible involvement of apoptosis- and stress-related responses OL-ZnONPs.

#### 3.4.4 Compound–target–pathway network analysis

While LC-MS/MS analysis quantified the abundance of eleven phenolic compounds in OL extract, network pharmacology was constructed to provide additional information on their predicted target and pathway associations. The compound-target-pathway network (Fig. 10a) comprised 901 nodes, including 12 components (11 identified phenolic compounds and ZnO), 801 target genes, and 89 enriched pathways, interconnected by 5160 edges. The multi-component, multi-target characteristics, and multi-pathways of olive leaf extract represent recognized advantage of herbal medicines, in contrast to the major limitation of conventional single-target chemotherapies. Owing to the network's complexity, degree centrality analysis was applied to simplify visualization and highlight the most highly connected nodes (Fig. 10b).

**Fig. 10:**
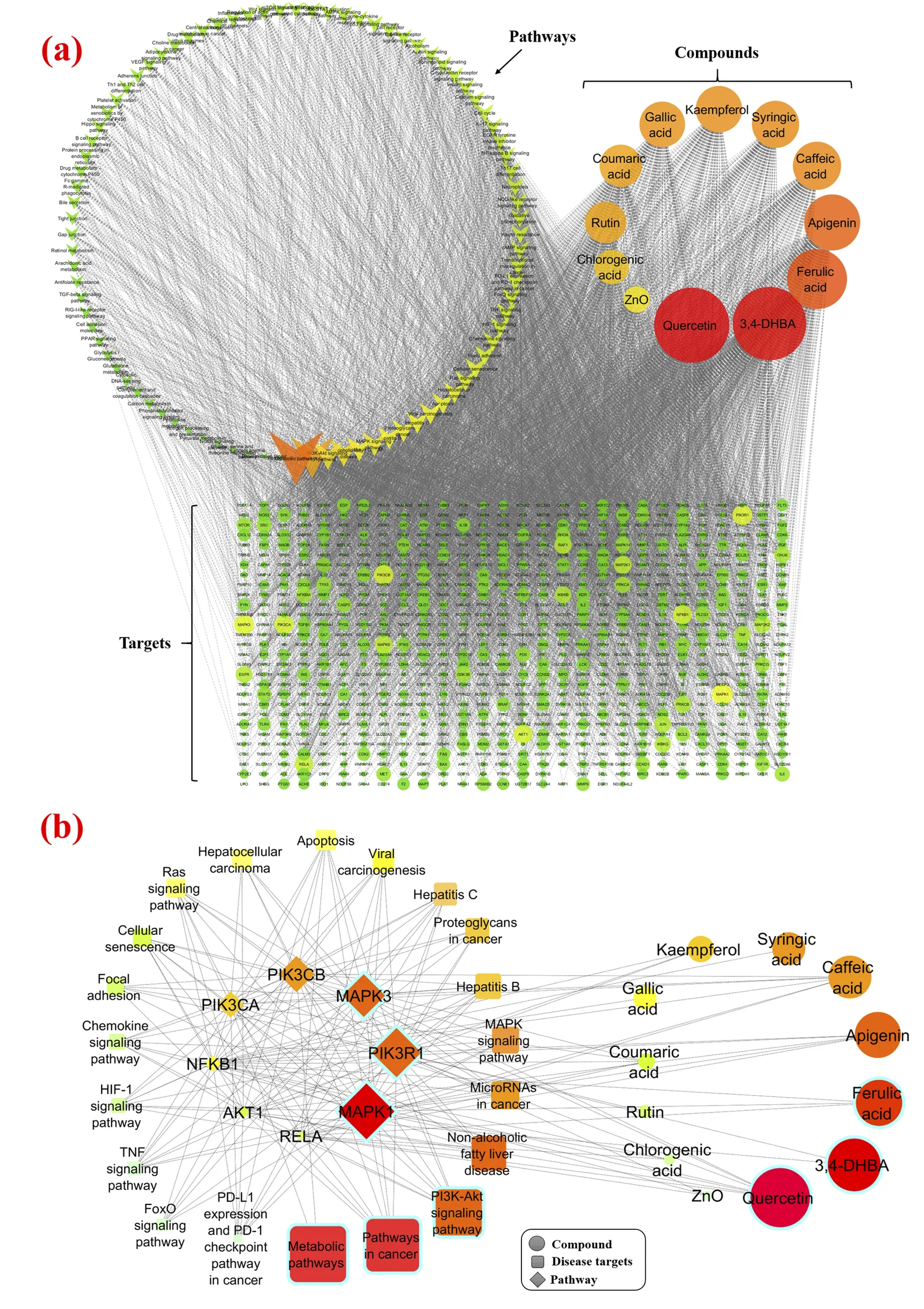
(a) Full network showing all compound–target–pathway interactions. (b) Filtered network based on degree centrality, highlighting key nodes.

Interestingly, quercetin, 3,4-dihydroxybenzoic acid (3,4-DHBA), and ferulic acid exhibited the highest compound degree values, identifying them as key “hub compounds” (Table 6). The hub targets identified included MAPK1, PIK3R1, and MAPK3, while significantly enriched pathways involved metabolic pathways, pathways in cancer, and the PI3K–Akt signaling pathways (Table 6).

**Table 6:** The top-ranked compounds, targets, and pathways associated with HCC disease

Classification	No.	Name	Degree	ClosenessCentrality	BetweennessCentrality
Core components	1	Quercetin	300	0.44932601	0.19992454
	2	3,4-DHBA	292	0.44576523	0.17278844
	3	Ferulic acid	230	0.41879944	0.13727042
	4	Apigenin	212	0.41227668	0.09395785
	5	Caffeic acid	176	0.39946738	0.0658684
	6	Syringic acid	171	0.39630119	0.0914828
	7	Kaempferol	168	0.3956044	0.04676845
	8	Gallic acid	164	0.39491005	0.066989
	9	Coumaric acid	149	0.3887689	0.05229478
	10	Rutin	145	0.38809832	0.03024326
	11	Chlorogenic acid	113	0.37578288	0.0427944
	12	ZnO	75	0.36363636	0.05895841
Core targets	1	MAPK1	50	0.43269231	0.0084824
	2	PIK3R1	46	0.44117647	0.00894425
	3	MAPK3	46	0.38071066	0.00415643
	4	PIK3CB	44	0.42213884	0.00611775
	5	PIK3CA	43	0.39929015	0.00499436
	6	NFKB1	42	0.47770701	0.01337698
	7	AKT1	41	0.42213884	0.00482835
	8	RELA	38	0.43774319	0.0086132
	9	MAP2K1	36	0.36585366	0.00253668
	10	MAPK8	36	0.45045045	0.00768043
	11	RAF1	35	0.35856574	0.00197666
	12	JUN	31	0.46391753	0.00903189
	13	MAP2K2	31	0.35742653	0.00190167
	14	IKBKB	31	0.36824877	0.00268607
	15	EGFR	31	0.46343975	0.00697674
	16	PRKCB	30	0.39787798	0.00316125
	17	IKBKG	30	0.41705283	0.00462694
	18	TNF	30	0.42372881	0.00477453
	19	CHUK	28	0.38927336	0.00265251
	20	PRKCA	27	0.39508341	0.00285164
Core pathways	1	Metabolic pathways	202	0.40268456	0.09650615
	2	Pathways in cancer	145	0.38643195	0.03549156
	3	PI3K-Akt signaling pathway	84	0.36481557	0.0104853
	4	Non-alcoholic fatty liver disease	81	0.36452005	0.01656381
	5	MicroRNAs in cancer	66	0.36043252	0.01002331
	6	MAPK signaling pathway	65	0.35899481	0.00757361
	7	Hepatitis B	60	0.35728464	0.0041908
	8	Proteoglycans in cancer	58	0.35643564	0.0043773
	9	Hepatitis C	56	0.35671819	0.00520952
	10	Viral carcinogenesis	55	0.35728464	0.00716773
	11	Apoptosis	53	0.35502959	0.0057719
	12	Hepatocellular carcinoma	52	0.35502959	0.00321496
	13	Ras signaling pathway	50	0.3539127	0.00363022
	14	Cellular senescence	50	0.35419126	0.00387964
	15	Focal adhesion	47	0.35197497	0.00315825
	16	Chemokine signaling pathway	47	0.35252644	0.00383874
	17	HIF-1 signaling pathway	45	0.35447026	0.00321004
	18	TNF signaling pathway	44	0.35252644	0.00388105
	19	FoxO signaling pathway	44	0.35252644	0.00269313
	20	PD-L1 expression and PD-1 checkpoint pathway in cancer	43	0.35142522	0.00317015

These network characteristics suggest that the predicted biological associations of the OL phytochemicals are not determined solely by their measured abundance, but also by the number and distribution of their predicted target associations. Network topology implies that quercetin and 3,4-DHBA act as primary bioactive drivers, contributing to the ZnO to modulate central hub targets obtained from the PPI network, such as TP53, TNF, and AKT1. This multi-component, multi-target mechanism in addition to the KEGG and GO enrichment results, provides a complementary systems-level context for interpreting the experimental findings.

Altogether, the network pharmacology findings provide mechanistic context for the observed anticancer activity of OL-ZnONPs and are consistent with the involvement of the associated olive-leaf phytochemicals and ZnO-based nanostructure. However, the present study does not allow quantitative separation of the individual contributions of the ZnO component and the associated phytochemicals. Future studies using size-matched bare ZnO nanoparticles with comparable physicochemical properties are therefore warranted to evaluate the contribution of the phytochemical constituents to the observed anticancer activity.

### 3.5 Molecular docking analysis

Protein–ligand interactions were analyzed using PoseView, which converts three-dimensional protein–ligand complexes into chemically intuitive two-dimensional diagrams based on standard drawing conventions. Molecular docking was performed using the available crystallographic structures of several cancer-related proteins, including AKT1 (PDB ID: 6HHG), TP53 (PDB ID: 8EOM), TNF-α (PDB ID: 2AZ5), interleukin-6 (PDB ID: 8QY5), caspase-3 (PDB ID: 3KJF), and caspase-9 (PDB IDs: 1NW9 and 3V3K). However, consistent docking results, based on the obtained binding affinity scores and interpretable two-dimensional interaction diagrams, were obtained only for AKT1, TP53, and caspase-3; therefore, subsequent docking analyses focused exclusively on these three targets. Directional protein–ligand interactions are represented as dashed lines, whereas hydrophobic interactions are represented by spline-based highlights corresponding to hydrophobic ligand regions and their interacting residues. Two-dimensional layouts were generated using the 2Ddraw library, and intermolecular interactions were assigned based on an internal model relying on atom-type classification and geometric criteria. For each ligand, the final pose was selected using a hierarchical strategy prioritizing the lowest binding energy and the presence of key interactions, including hydrogen bonding with residues such as Tyr150B, Tyr157B, Leu156B, Ser267B, and Tyr175A, as illustrated in Fig. 11.

**Fig. 11:**
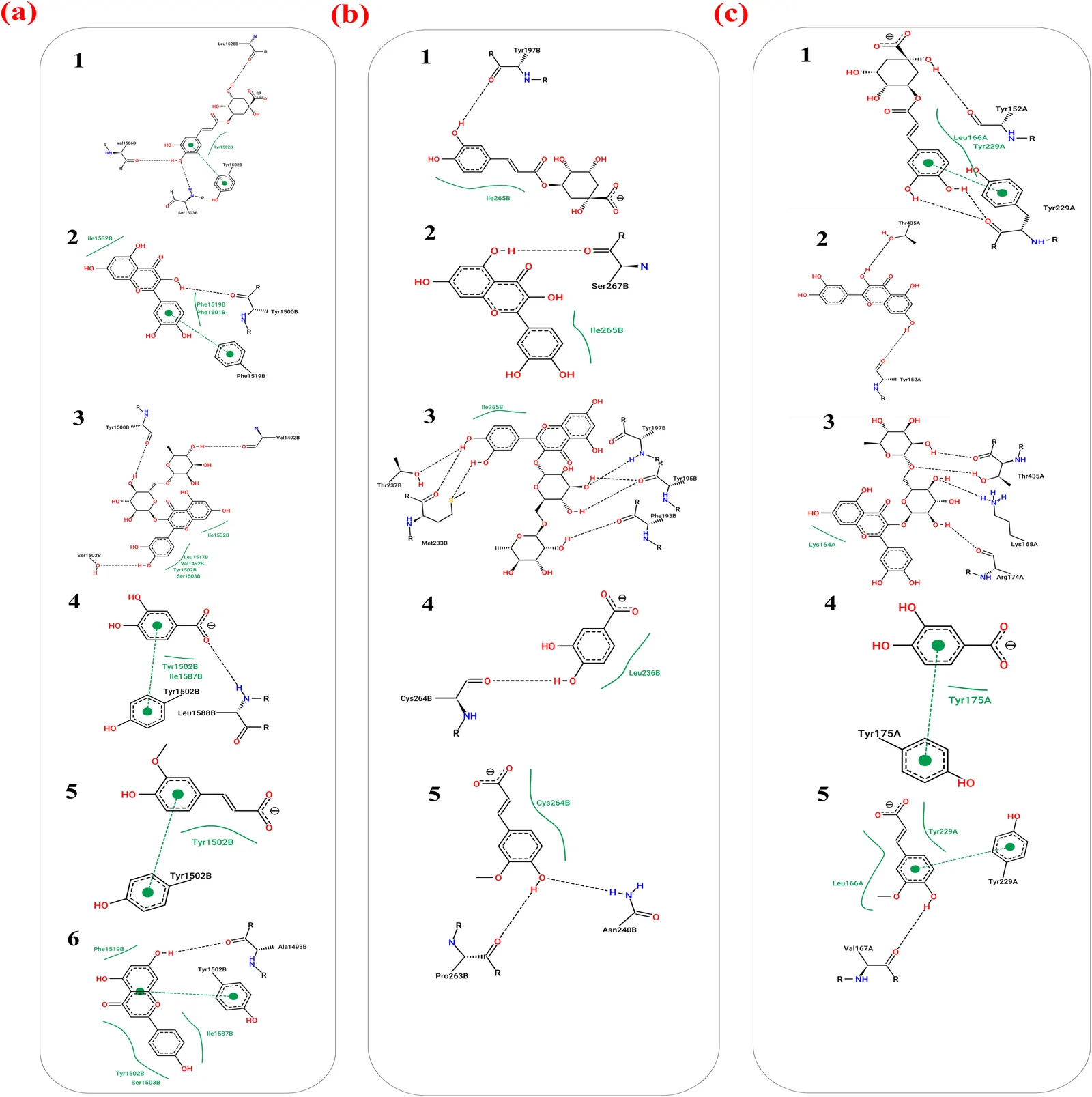
Two-dimensional ligand–protein interaction diagrams showing molecular docking of four predominant compounds: (1) chlorogenic acid, (2) quercetin, (3) rutin, (4) 3,4-dihydroxybenzoic acid, (5) ferulic acid, and (6) apigenin against three target proteins: (a) TP53 (PDB ID: 8EOM), (b) caspase-3 (PDB ID: 3KJF), and (c) AKT1 (PDB ID: 6HHG).

Docking simulations were conducted using rutin, 3,4-dihydroxybenzoic acid, quercetin, chlorogenic acid, ferulic acid, and apigenin, with ligand structures retrieved from PubChem and defined by their corresponding SMILES notations (Table S2). Binding affinities and interaction patterns for AKT1, TP53, and caspase-3 are summarized in Table 7 and illustrated in Fig. 11.

**Table 7:** Calculated binding affinities (kcal mol^−1^) of the active compounds against key cancer-related proteins

Protein	Ligand	Calculated affinity (kcal mol^−1^)
AKT1	Quercetin	−9.278
	3,4-Dihydroxybenzoic acid	−5.976
	Rutin	−11.381
	Chlorogenic acid	−9.192
	Ferulic acid	−6.820
TP53	Quercetin	−5.638
	3,4-Dihydroxybenzoic acid	−4.110
	Rutin	−2.631
	Chlorogenic acid	−5.211
	Ferulic acid	−5.290
	Apigenin	−5.391
CASP3	Quercetin	−6.004
	3,4-Dihydroxybenzoic acid	−4.674
	Rutin	−7.191
	Chlorogenic acid	−5.605
	Ferulic acid	−4.830

Rutin and quercetin showed strong interactions with AKT1 and caspase-3, consistent with *in vitro* potential involvement in apoptosis-related signaling and migration inhibition by phenolic compounds, such as ferulic acid and chlorogenic acid.^60^ Rutin has also been reported to regulate several signaling pathways, including PI3K/Akt, MAPK, and NF-κB, in agreement with KEGG and GO enrichment results. In addition, quercetin exhibited strong binding to TP53 (−5.638 kcal mol^−1^), compatible with previous reports showing that quercetin inhibited HepG-2 cell proliferation through cell cycle arrest and DNA fragmentation and was associated with p53 phosphorylation without transcriptional up-regulation of p53 mRNA.^61^ Moreover, apigenin displayed favorable binding affinity (−5.391 kcal mol^−1^). Overall, the docking results identified plausible interactions between selected olive-leaf phytochemicals and AKT1, TP53, and caspase-3, providing computational support for their potential involvement in apoptosis-related signaling. These findings align with network pharmacology and KEGG and GO enrichment analyses, suggesting that selective phytoconstituents of OLE and ZnO component in OL-ZnO nanoparticles exert their anticancer activity through a multi-pathway mechanism.

Nevertheless, it should be noted that network pharmacology and molecular docking provide predictive and hypothesis-generating insights into the potential multi-target interactions of selected olive-leaf phytoconstituents. These computational approaches do not account fully for cellular context, protein flexibility, solvent effects, or the complexity of the nanoparticle–cell interface. Therefore, the predicted protein-ligand interactions should not be interpreted as evidence and the proposed mechanisms remain require further *in vivo* experimental validation through future studies.

## 4 Conclusion

This study established an integrated green nanotechnology and analytical framework for valorizing olive leaf agricultural residues into zinc oxide nanoparticles (OL-ZnONPs) with potent anticancer activity and a favorable selectivity profile toward HepG-2 cells. The biosynthesized OL-ZnONPs displayed a well-defined hexagonal wurtzite crystalline structure (15.47 nm average crystallite size), favorable colloidal stability (zeta potential −23.7 mV), and distinct morphology confirmed by TEM and SEM-EDX. LC-ESI-MS/MS-MRM profiling was performed on 70% olive leaf ethanolic extract in negative ionization mode using 21 standard compounds. Eleven phenolic compounds were identified and quantified, with rutin exhibiting the highest abundance (354.15 µg g^−1^), followed by 3,4-DHBA (78.21 µg g^−1^) and quercetin (45.75 µg g^−1^). Additional identified compounds included gallic acid, caffeic acid, coumaric acid, kaempferol, ferulic acid, syringic acid, apigenin, and chlorogenic acid. The analytical method demonstrated excellent linearity (*R*^2^ > 0.995) and high sensitivity (LOD 0.0446–0.0978 µg g^−1^; LOQ 0.1353–0.2964 µg g^−1^).

Biological assays revealed that OL-ZnONPs exerted potent cytotoxicity against HepG-2 (human hepatoma), MDA (human breast cancer), and HCT-116 (human colon cancer) cells, the strongest selectivity observed toward HepG-2 cells (IC_50_ = 0.344 µg mL^−1^; SI = 5.279), compared with the OL extract (IC_50_ = 15.00 ± 0.645 µg mL^−1^; SI 1.33) and 5-FU (IC_50_ = 3.383 ± 1.349 µg mL^−1^; SI 0.943). Mechanistic studies showed apoptosis-related changes characterized by increased intracellular levels of total caspase-3 and caspase-9, reduced TNF-α and IL-6 levels, nuclear alterations consistent with apoptosis, and inhibition of HepG-2 cell migration. Computational analyses using network pharmacology and molecular docking identified quercetin, 3,4-DHBA, and ferulic acid as high-degree hub compounds in the compound–target–pathway network, together with highly connected target nodes including AKT1, TP53, and CASP3, and predicted favorable interactions between selected phytochemicals and these targets across cancer-related, apoptosis-, PI3K–Akt-, and cellular stress-response pathways. These computational findings provide mechanistic context for the experimental observations but remain predictive and hypothesis-generating, as they do not establish a direct or synergistic interaction between the phytochemicals and the ZnO core.

Overall, OL-ZnONPs combine the effects of ZnO with the bioactive components of olive leaf extract, with changes in caspase levels, p53 expression, and survival-related pathways observed following treatment. This work illustrates how sustainable valorization and advanced analytical innovation can transform agricultural residues into high-value biomaterials with translational potential for cancer therapy. Importantly, this work aligns with the UN Sustainable Development Goals: SDG 3 (good health and well-being), SDG 9 (Industry, innovation and infrastructure), SDG 12 (responsible consumption/production), and SDG 13 (climate action) by converting biowaste into targeted anticancer therapeutics through green nanotechnology, thereby reducing agricultural waste, lowering the carbon footprint, and promoting sustainable industrial innovation.

Further studies should focus on optimizing OL-ZnONPs through nanocarrier-based formulations to improve and delivery strategies, as well as isolating individual olive leaf bioactive compounds to better define their specific contributions and further clarify the underlying mechanisms. Comparative studies using size-matched bare ZnO nanoparticles with comparable physicochemical properties are also warranted to distinguish the contribution of the ZnO core from that of the associated phytochemical constituents and to determine whether their combined effects are additive or synergistic. In addition, comprehensive *in vivo* studies addressing pharmacokinetics, biosafety, and therapeutic efficacy in preclinical hepatocellular carcinoma models will be essential to further assess the translational relevance of OL-ZnONPs.

## Author contributions

Conceptualization, Seham S. El-Hawary, Mohamed El Raey, and Yasmin Mounir Mohamaden; methodology, Yasmin Mounir Mohamaden, Yousra A. El-Maradny, and Mohamed El Raey; software, Yasmin Mounir Mohamaden and Yousra A. El-Maradny; validation, Yasmin Mounir Mohamaden and Yousra A. El-Maradny; formal analysis, Yasmin Mounir Mohamaden and Yousra A. El-Maradny; investigation, Yasmin Mounir Mohamaden, Yousra A. El-Maradny, and Mohamed El Raey; resources, Yasmin Mounir Mohamaden, Yousra A. El-Maradny, and Mohamed El Raey; data curation, Yasmin Mounir Mohamaden and Yousra A. El-Maradny; writing – original draft Preparation, Yasmin Mounir Mohamaden and Yousra A. El-Maradny; writing – review and editing, Seham S. El-Hawary, Mohamed A. Rabeh, Mohammad D. AL-Zaid, Mohamed El Raey, Esmail M. El-Fakharany, Amira Safwat El Senousy, Samar M. Bassam, Yousra A. El-Maradny, and Yasmin Mounir Mohamaden; visualization, Yasmin Mounir Mohamaden and Yousra A. El-Maradny; supervision, Seham S. El-Hawary, Mohamed El Raey, Amira Safwat El Senousy, and Samar M. Bassam. All authors have read and agreed to the published version of the manuscript.

## Conflicts of interest

There are no conflicts to declare.

## Abbreviations

DLS: Dynamic light scattering
EC_50_: Half-maximal effective concentration
EDX: Energy dispersive X-ray spectroscopy
ELS: Electrophoretic light scattering
FBS: Fetal bovine serum
FT-IR: Fourier transform infrared
5-FU: 5-Fluorouracil
FWHM: Full width at half maximum
GO: Gene enrichment
IC_50_: Half-maximal inhibitory concentration
ICDD: International Centre for Diffraction Data
IL-6: Interleukin-6
JCPDS: The Joint Committee on Powder Diffraction Standards
KEGG: Kyoto Encyclopedia of Genes and Genomes
LC-ESI-MS/MS: Liquid chromatography–electrospray ionization–tandem mass spectrometry
LOD: Limit of detection
LOQ: Limit of quantification
MRM: Multiple-reaction monitoring
OL: Olive leaves
OL-ZnONPs: Olive leaves extract-mediated zinc oxide nanoparticles
PDI: Polydispersity index
PI: Propidium iodide
ROS: Reactive oxygen species
SAED: Selected area electron diffraction
SDG: Sustainable development goals
SEM: Scanning electron microscope
SI: Selectivity index
TEM: Transmission electron microscope
TNF-α: Tumor necrosis factor-α
XRD: X-Ray diffraction
MDPI: Multidisciplinary Digital Publishing Institute
DOAJ: Directory of open access journals
TLA: Three-letter acronym
LD: Linear dichroism

## Supplementary Material

RA-016-D6RA01605G-s001

## Data Availability

Data is provided within the manuscript. In addition, the supporting data has been provided as part of the supplementary information (SI). Supplementary information: Fig. S1 (global cancer burden estimates, GLOBOCAN 2024, released in the 2026. Fig. S2 (calibration curves of 10 phenolic standards quantified *via* LC-ESI-MS/MS in negative ionization MRM mode), Fig. S3 (cell viability and cytotoxicity percentage of OL-ZnONPs, OL extracts, and 5-FU on MDA, HepG-2, and HCT-116 cell lines), Table S1 (dose-response data of the OL-ZnONPs, extracts, and 5-FU on HSF cells), and Table S2 (SMILES notations and PubChem IDs of ligands used for molecular docking). See DOI: https://doi.org/10.1039/d6ra01605g.
