# Supplementary material for: Sustainable nanomedicine from olive leaf waste: biosynthesized ZnONPs with mechanistic validation against hepatocellular carcinoma
Source: RSC Adv. 2026 Oct 6;16(46):60479–504. doi: 10.1039/d6ra01605g (PMC13640615; doi:10.1039/d6ra01605g)
Supplement: Supplementary file 1 [file RA-016-D6RA01605G-s001.pdf]

Supplementary Information (SI) for RSC Advances.
This journal is © The Royal Society of Chemistry

The Supplementary file of "**Sustainable Nanomedicine from Olive Leaf Waste: Biosynthesized ZnONPs with Mechanistic Validation Against Hepatocellular Carcinoma**"

Yasmin Mounir Mohamaden [a,b,*], Seham S. El-Hawary [a,*], Esmail M. El-Fakharany [c], Yousra A. El-Maradny [c], Mohamed El Raey [d], Amira Safwat El Senousy [a], Mohamed A. Rabeh [e], Mohammad D. AL-Zaid [f], Samar M. Bassam [g]

* Email: corresponding author 1 yasmin.m.mohamaden@pharma.cu.edu.eg, Yasmin.mounir@aast.edu, yasmin.m0unir.nm@gmail.com

* Email: corresponding author 2 seham.elhawary@pharma.cu.edu.eg

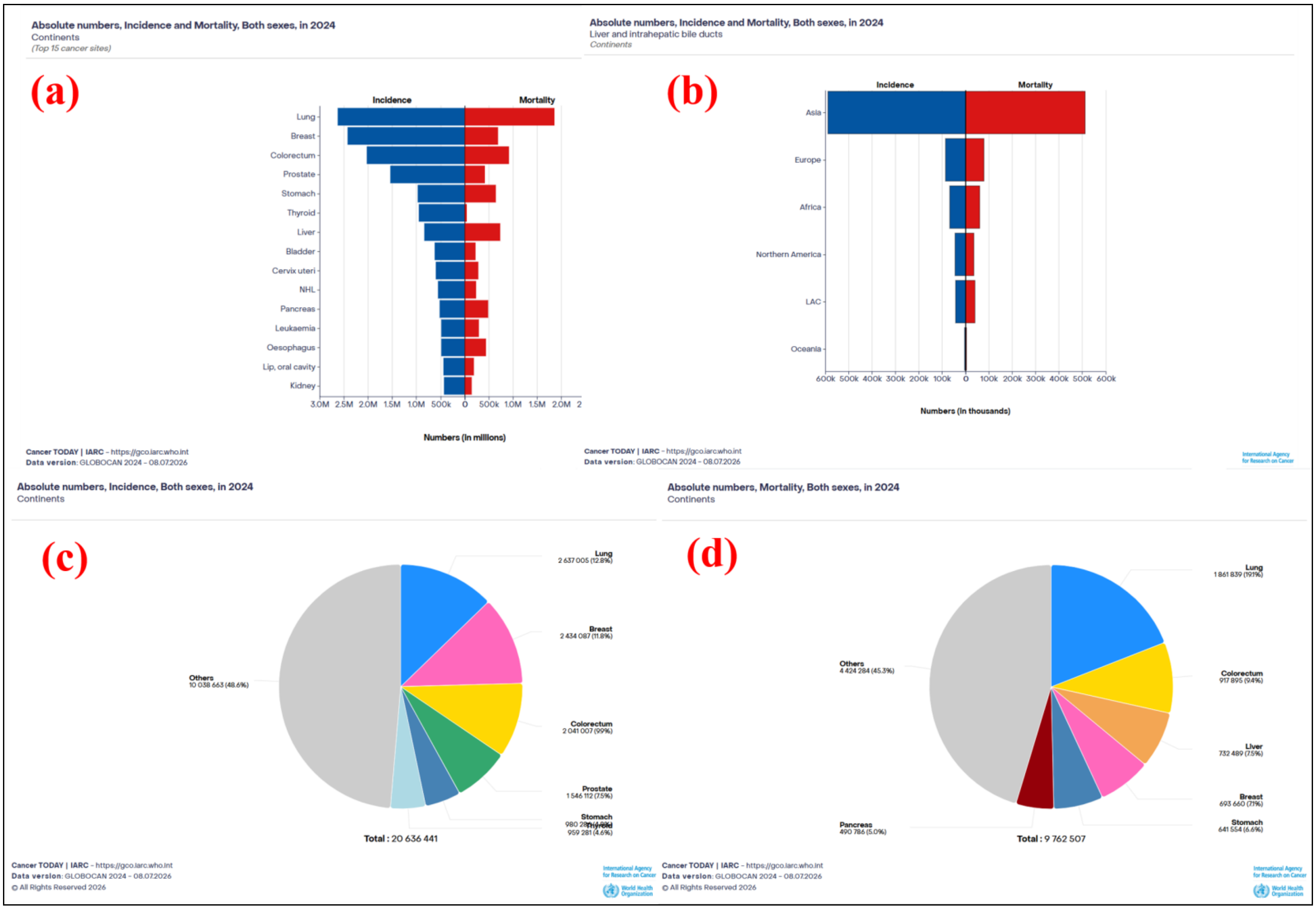

Figure S1. Global cancer burden estimates based on GLOBOCAN 2024 data, released in the 2026 release: **(a)** Comparative bar graph showing the absolute numbers of incidence *vs.* mortality for the top 15 cancer sites, indicating a high fatality ratio. **(b)** Regional breakdown of liver and intrahepatic bile duct cancer statistics, displaying incidence and mortality figures across different continents. **(c)** Pie chart illustrating the distribution of new cancer cases (incidence) across major tumor sites, where liver cancer ranks sixth globally. **(d)** Pie chart depicting the proportion of

cancer-related deaths, highlighting liver cancer as the third leading cause of mortality worldwide. **Source:** International Agency for Research on Cancer (IARC), Cancer Today [1, 2].

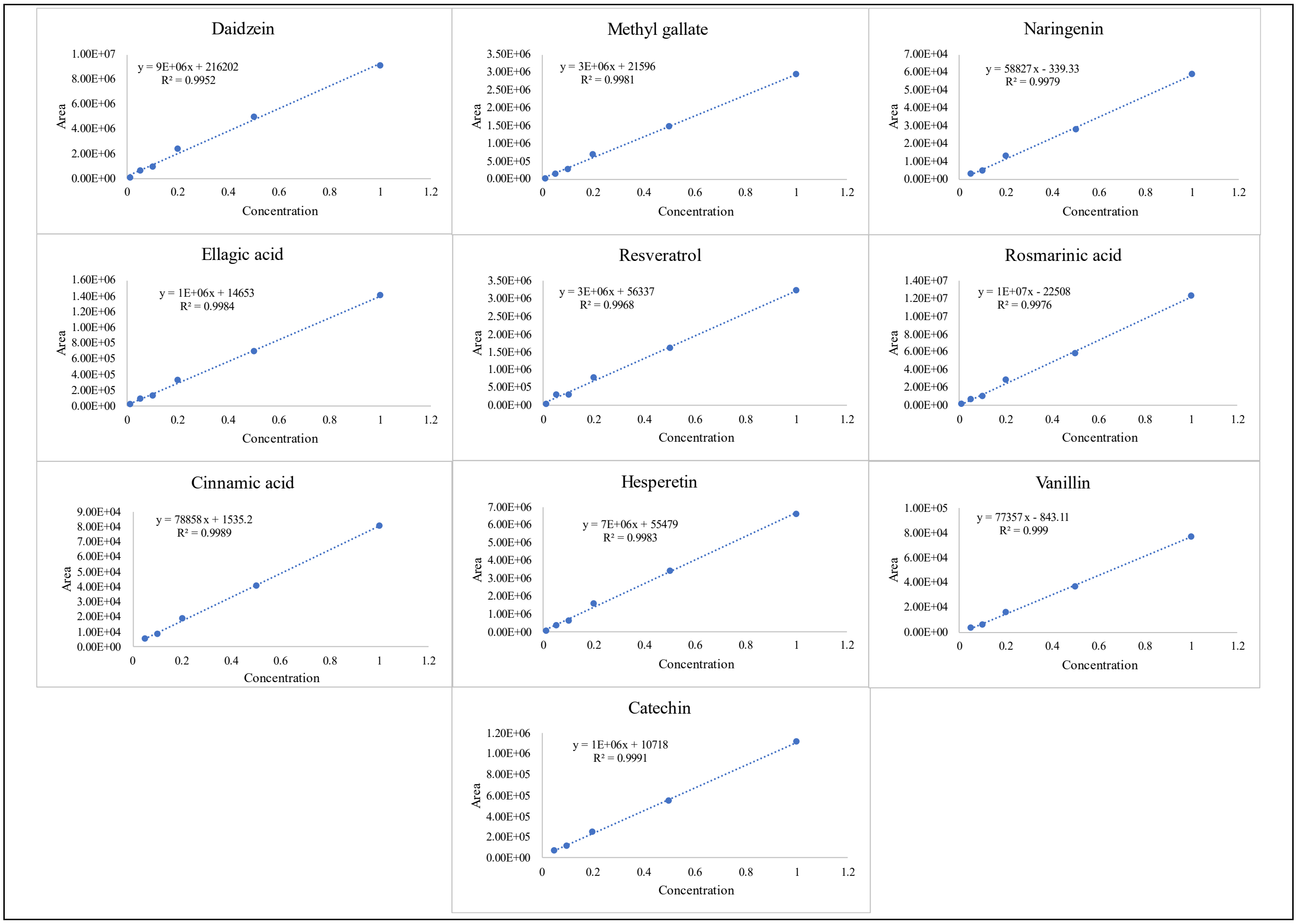

Figure S2. Calibration curves of 10 phenolic standards quantified *via* LC–ESI–MS/MS in negative ionization MRM mode. Each curve represents the linear relationship between concentration and peak area, with corresponding regression equations and R^2 values, indicating high analytical accuracy and reproducibility.

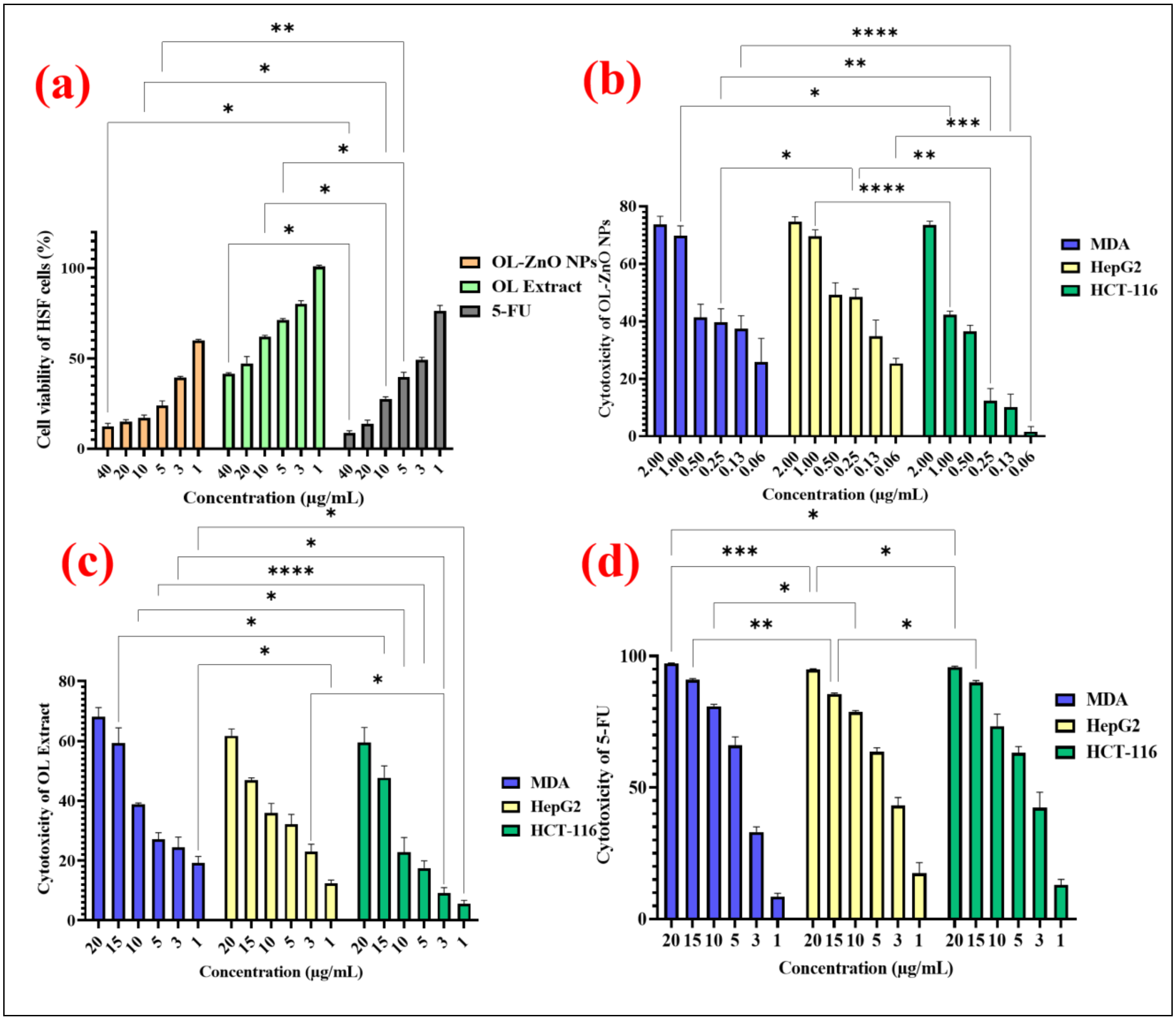

Figure S3. Cell viability of OL-ZnONPs, OL extracts, and 5-FU **(a)**, and cytotoxicity percentage of OL-ZnONPs **(b)**, OL extracts **(c)**, and 5-FU **(d)** on MDA, HepG2, and HCT-116 cell lines. Data are presented as mean ± SD (n = 3). Statistical significance is indicated as follows: ****$p < 0.0001$; ***$p < 0.001$; **$p < 0.01$; *$p < 0.05$.

Table S1. Dose-response analysis of the OL-ZnONPs, extracts, and 5-FU on human skin fibroblast (HSF) cell line.

**Concentration (µg/mL)**	**Tested samples (µg/mL)**		
	OL-ZnONPs	Olive leaf extract	5-Flurouracil
**40**	12.43 ± 1.55	41.45 ± 0.62	8.63 ± 1.27
**20**	14.84 ±1.24	47.22 ± 3.83	13.79 ± 1.97
**10**	16.92 ± 1.71	62.06 ± 0.72	27.59 ±1.16
**5**	23.83 ± 2.69	71.24 ± 0.88	39.70 ± 2.61
**2.5**	39.40 ± 0.62	80.19 ± 1.76	49.23 ± 1.34
**1.25**	59.90 ± 0.67	100.88 ± 0.72	76.40 ± 2.95
**EC_{50}**	**1.816 ± 0.8244**	**20.02 ± 1.6497**	**3.189 ± 0.9677**

*OL-ZnONPs, Olive leaves extract-mediated zinc oxide nanoparticles; EC_{50}, half-maximal effective concentration

Table S2. SMILES notations and PubChem identifiers of the ligands used for molecular docking

Ligand	SMILES
**Chlorogenic acid**	C1[C@H]([C@H]([C@@H](C[C@@]1(C(=O)O)O)OC(=O)/C=C/C2=CC(=C(C=C2)O)O)O)O
**Quercetin**	C1=CC(=C(C=C1C2=C(C(=O)C3=C(C=C(C=C3O2)O)O)O)O)O
**Rutin**	C[C@H]1[C@@H]([C@H]([C@H]([C@@H](O1)OC[C@@H]2[C@H]([C@@H]([C@H]([C@@H](O2)OC3=C(OC4=CC(=CC(=C4C3=O)O)O)C5=CC(=C(C=C5)O)O)O)O)O)O)O)O
**3,4-dihydroxybenzoic acid**	C1=CC(=C(C=C1C(=O)O)O)O
**Ferulic acid**	COC1=C(C=CC(=C1)/C=C/C(=O)O)O
**Apigenin**	C1=CC(=CC=C1C2=CC(=O)C3=C(C=C(C=C3O2)O)O)O

References

1. I. A. f. R. o. Cancer, Global Cancer Observatory: Cancer Today - Absolute numbers, Incidence and Mortality, Both sexes, in 2022, https://gco.iarc.fr/today/en/dataviz/bars?mode=cancer&key=total&cancers=15&group_populations=1&types=0_1&sort_by=value1, (accessed 26 December 2025).
2. I. A. f. R. o. Cancer, Global Cancer Observatory: Cancer Today - Absolute numbers, Mortality, Both sexes, in 2022, https://gco.iarc.fr/today/en/dataviz/pie?mode=cancer&key=total&cancers=11&sort_by=value0&group_populations=1&types=1&populations=903_904_905_908_909_935, (accessed 26 December 2025).
